# Epigenetic silencing of tumor suppressor candidate 3 confers adverse prognosis in early colorectal cancer

**DOI:** 10.18632/oncotarget.20950

**Published:** 2017-09-15

**Authors:** Elke Burgermeister, Patrick Höde, Johannes Betge, Tobias Gutting, Andreas Merkel, Wen Wu, Marc Tänzer, Maximilian Mossner, Daniel Nowak, Julia Magdeburg, Felix Rückert, Carsten Sticht, Katja Breitkopf-Heinlein, Nadine Schulte, Nicolai Härtel, Sebastian Belle, Stefan Post, Timo Gaiser, Barbara Ingold Heppner, Hans-Michael Behrens, Christoph Röcken, Matthias P.A. Ebert

**Affiliations:** ^1^ Department of Medicine II, University Hospital Mannheim, Medical Faculty Mannheim, Heidelberg University, Mannheim, Germany; ^2^ Department of Medicine II, Klinikum rechts der Isar, Technische Universität München, Munich, Germany; ^3^ Department of Medicine III, University Hospital Mannheim, Medical Faculty Mannheim, Heidelberg University, Mannheim, Germany; ^4^ Department of Surgery, University Hospital Mannheim, Medical Faculty Mannheim, Heidelberg University, Mannheim, Germany; ^5^ Center for Medical Research (ZMF), Medical Faculty Mannheim, Heidelberg University, Mannheim, Germany; ^6^ Institute of Pathology, University Hospital Mannheim, Medical Faculty Mannheim, Heidelberg University, Mannheim, Germany; ^7^ Institute of Pathology, University Hospital Charite, Berlin, Germany; ^8^ Institute of Pathology, Christian-Albrechts University, Kiel, Germany

**Keywords:** TUSC3, colorectal cancer, prognosis, glycosylation, growth factor receptor

## Abstract

Colorectal cancer (CRC) is a biologically and clinically heterogeneous disease. Even though many recurrent genomic alterations have been identified that may characterize distinct subgroups, their biological impact and clinical significance as prognostic indicators remain to be defined. The tumor suppressor candidate-3 (*TUSC3/N33*) locates to a genomic region frequently deleted or silenced in cancers. TUSC3 is a subunit of the oligosaccharyltransferase (OST) complex at the endoplasmic reticulum (ER) which catalyzes bulk N-glycosylation of membrane and secretory proteins. However, the consequences of TUSC3 loss are largely unknown. Thus, the aim of the study was to characterize the functional and clinical relevance of TUSC3 expression in CRC patients’ tissues (*n*=306 cases) and cell lines. *TUSC3* mRNA expression was silenced by promoter methylation in 85 % of benign adenomas (*n*=46 cases) and 35 % of CRCs (*n* =74 cases). Epidermal growth factor receptor (EGFR) was selected as one exemplary ER-derived target protein of TUSC3-mediated posttranslational modification. We found that TUSC3 inhibited EGFR-signaling and promoted apoptosis in human CRC cells, whereas TUSC3 siRNA knock-down increased EGFR-signaling. Accordingly, in stage I/II node negative CRC patients (*n*=156 cases) loss of TUSC3 protein expression was associated with poor overall survival. In sum, our data suggested that epigenetic silencing of TUSC3 may be useful as a molecular marker for progression of early CRC.

## INTRODUCTION

Recently, a vast number of different reoccurring genomic alterations in colorectal cancer (CRC) have been identified, including mutations, copy number alterations or epigenetic modifications [1, 2]. However, only a minority of molecular alterations in CRC have been studied for their biological mode of action and even less for their clinical relevance. Strikingly, clinical decision-making is to date still mainly based on the anatomical stage of the disease and histomorphological parameters [3], while almost no molecular marker is routinely used for therapeutic decisions. Therefore, detailed studies of recurrent molecular alterations, their mode of action and their impact as prognostic or predictive markers are warranted.

Signaling through growth factor receptors is a major contributor to cell proliferation and survival in CRC [4, 5]. Activation may occur through mutations which are frequently found already in early stages of carcinogenesis [6]. Receptor activities, including insulin-like growth factor and ErbB receptors, may, however, also be regulated through changes in N-glycosylation, including alterations in the composition of branched sugar residue patterns. This so far under-recognized mechanism in carcinogenesis has been associated with cancer progression and metastasis [7]. For example, the epidermal growth factor receptor (EGFR/Her1) needs N-glycosylation to be functional [8, 9]. In contrast, complex carbohydrate adducts (such as gangliosides) or enzymes which modify terminal sugar residues (such as sialidases) may inhibit ErbB signaling through steric hindrance [10, 11]. Aberrant or defective N-glycosylation of growth factor receptors may therefore add a new level of regulation to human carcinogenesis [7]. However, the link between enzymes that catalyze N-glycosylation in the endoplasmic reticulum (ER) and early carcinogenesis is so far unknown.

Here, we studied expression, function and clinical significance of the tumor suppressor candidate-3 (TUSC3/N33) [12, 13] in CRC cells and tissues. TUSC3 is one subunit of the oligosaccharyl-transferase (OST) multiprotein complex at the ER-membrane, proposing a function for TUSC3 in the initial steps of protein N-glycosylation [14, 15]. We demonstrate that TUSC3 is epigenetically silenced already in benign adenomas, and loss of TUSC3 protein expression correlates with poor survival in early stages of CRC. In human CRC cells, TUSC3 inhibited EGFR signaling, an exemplary target protein of OST-mediated N-glycosylation in the ER, thus providing one potential mechanism by which loss of TUSC3 contributes to progression of CRC.

## RESULTS

### *TUSC3* is silenced by gene methylation early in human CRC tumorigenesis

**Figure 1:**
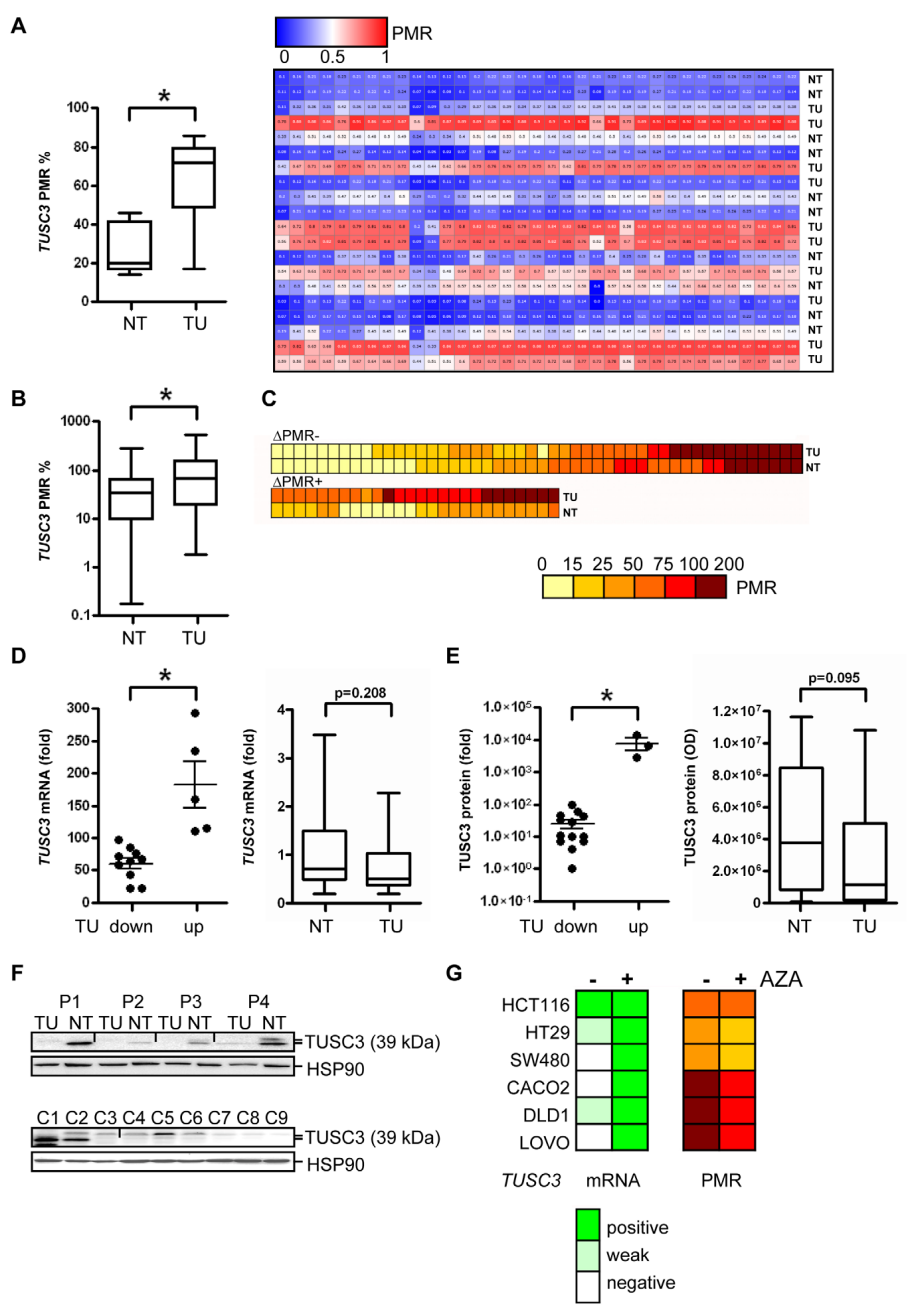
*TUSC3* is down-regulated in a large subgroup of CRC patients by epigenetic silencing **A.**, Validation of *TUSC3* promoter methylation in human CRC by next generation sequencing (NGS). DNA was extracted from CRC patients, bisulfite converted and sequenced comparing matched TU and NT tissue. Left panel: quantitative comparison of PMR values from TU *vs.* NT samples (*p = 0.0016; paired *t*-test, *n* = 10 cases), right panel: individual cases. **B.**-**C.**, Detection of *TUSC3* promoter methylation in human CRC by ML-PCR. DNA was extracted from CRC patients from TU and NT tissue. PCRs were performed, and the PMR values calculated and presented as color code. Comparison of TU and NT samples (*p < 0.0001, Wilcoxon signed rank test, *n* = 74 cases, B); detection of *TUSC3* methylation in both TU and NT samples (upper panel, C); differential *TUSC3* methylation in a subgroup of TU and NT samples (lower panel, C). **D.**, *TUSC3* mRNA expression is down-regulated in CRC. Total RNA was extracted, and CT-values were normalized to beta2-microglobulin (*B2M*) and calculated as -fold ± S.E. of TU compared to NT (mean of 4 healthy individuals) tissue (*p = 0.0007, Mann Whitney test, *n* = 15 cases, left panel). **E.**, Quantitative analyses of Western blots detecting endogenous TUSC3 protein in total tissue lysates from frozen TU and NT samples of CRC patients. O.D. values from bands in gels were normalized to HSP90 as a loading control and calculated as -fold ± S.E. (*p = 0.0098, Mann Whitney test, *n* = 17 cases, left panel). **F.**, Representative Western blots from total cell and tissue lysates are shown which detect a major band at 39 kDa for TUSC3 protein. Top panel: TU and matched NT samples from the same patients (P1-P4) were analyzed. Bottom panel: C1 = HEK293T cells transfected with TUSC3 plasmid, C2 = HEK293T transfected with FLAG-TUSC3 plasmid, C3 = HEK293T transfected with EV plasmid, C4 = SW480, C5 = HCT116, C6 = HT29, C7 = CACO2, C8 = LOVO, C9 = DLD1. **G.**, Detection of *TUSC3* promoter methylation (right panel) and mRNA expression (left panel) in human CRC cell lines by ML-PCR and RT-qPCR, respectively. After incubation of cells with and without the demethylation agent AZA (at 10 µM) for 3 days, DNA and total RNA were extracted. Color codes represent PMR for DNA methylation and scores for mRNA expression.

*TUSC3* locates to chromosome 8p22, a genomic region (S1) frequently deleted or epigenetically silenced in human cancers (including CRC, lung, prostate and breast) [12, 13]. First, we aimed to validate the presence of *TUSC3* methylation in CRC tissues, to study its prevalence and occurrence in the adenoma-carcinoma-sequence as well as putative associations with clinical factors. *TUSC3* methylation was confirmed by next generation sequencing (NGS) in a small series of matched non-tumor (NT) colon and tumor (TU) samples from CRC patients (Figure 1A). The percent methylation ratio (PMR) was higher in the TU compared with the NT tissue (TU 63.7±7.7 *vs*. NT 26.7±4.2, *p = 0.0016, paired *t*-test, *n* = 10 cases). A significant increase for *TUSC3* methylation was also observed in a larger cohort of CRC patients using MethyLight PCR (ML-PCR) (TU 106.8±13.5 *vs.* NT 45.9±6.0, *p < 0.0001, Wilcoxon signed rank test, *n* = 74 cases) (Figure 1B). Interestingly, *TUSC3* methylation was detected in both, TU and NT tissues of a patient subgroup, possibly caused by an age-related field effect, while 35 % of the samples (26 of 74) showed differential methylation of *TUSC3* (Figure 1C). Correlations of *TUSC3* methylation with clinical characteristics including age, gender, tumor location, pTNM-categories, grade (G) and mucinous subtype were not observed in this patient cohort (clinical information available from *n* = 64 cases, S2). Of note, a significant correlation (*p = 0.0068, Fisher exact test, *n* = 63 cases) between the combination of *APC* plus *TP53* mutations and *TUSC3* methylation (S3) was found in RanPlex CRC arrays, while there was no correlation of *TUSC3* methylation with *KRAS* or *BRAF* mutations alone. We also measured methylation of the *TUSC3* gene in patients with adenomas using ML-PCR. The overall PMR was significantly elevated in adenomas (AD) compared to matched normal colon (NC) tissue (S4).

These data confirmed that *TUSC3* is epigenetically silenced in a large subgroup of CRC patients, corroborating its role as a putative tumor suppressor. Furthermore, methylation in adenomas indicated that silencing of *TUSC3* is an early event in CRC carcinogenesis.

### *TUSC3* methylation is associated with down-regulation of *TUSC3* expression in CRC

We further studied the impact of *TUSC3* methylation on gene expression in tissue samples from CRC patients and in human CRC cell lines. Decreased *TUSC3* mRNA levels were detected by RT-qPCR analysis in the majority of CRC samples compared to NT colon tissue (TU 60.1±7.9 *vs.* NT 182.6±35.7, *p = 0.0007, Mann Whitney test, *n* = 15 cases) (Figure 1D). Accordingly, endogenous TUSC3 protein (isoform 1 and 2 of approx. 39 kDa) was not present in whole-tissue lysates from CRC compared to matched NT tissue (TU 25.2±7.5 *vs.* NT 7767±3256, *p = 0.0098, Mann Whitney test, *n* = 17 cases) (Figure 1E). Representative Western blots are displayed (Figure 1F, top panel). Low amount of endogenous TUSC3 protein was expressed in HCT116 cells, while it was undetectable in the other cell lines tested including the non-cancer cell line HEK293T (Figure 1F, bottom panel). Likewise, expression of *TUSC3* mRNA was found to be low or absent in a series of human CRC cell lines (CACO2, DLD1, HCT116, HT29, LOVO, SW480). When cells were treated with the methylation inhibitor 5-aza-(2-deoxy)-cytidine (AZA) for 3 days, *TUSC3* mRNA was re-increased in 5 out of 6 cell lines tested (except HCT116, Figure 1G, left panel). ML-PCR analysis corroborated *TUSC3* hypermethylation in the cell lines, whereas the PMR was reduced by 10 to 25 % upon treatment of cells with AZA (Figure 1G, right panel). These data suggested that *TUSC3* gene methylation is associated with loss of *TUSC3* mRNA and protein expression in CRC cell lines and patients.

### TUSC3 inhibits EGFR phosphorylation and signaling in human CRC cells

**Figure 2:**
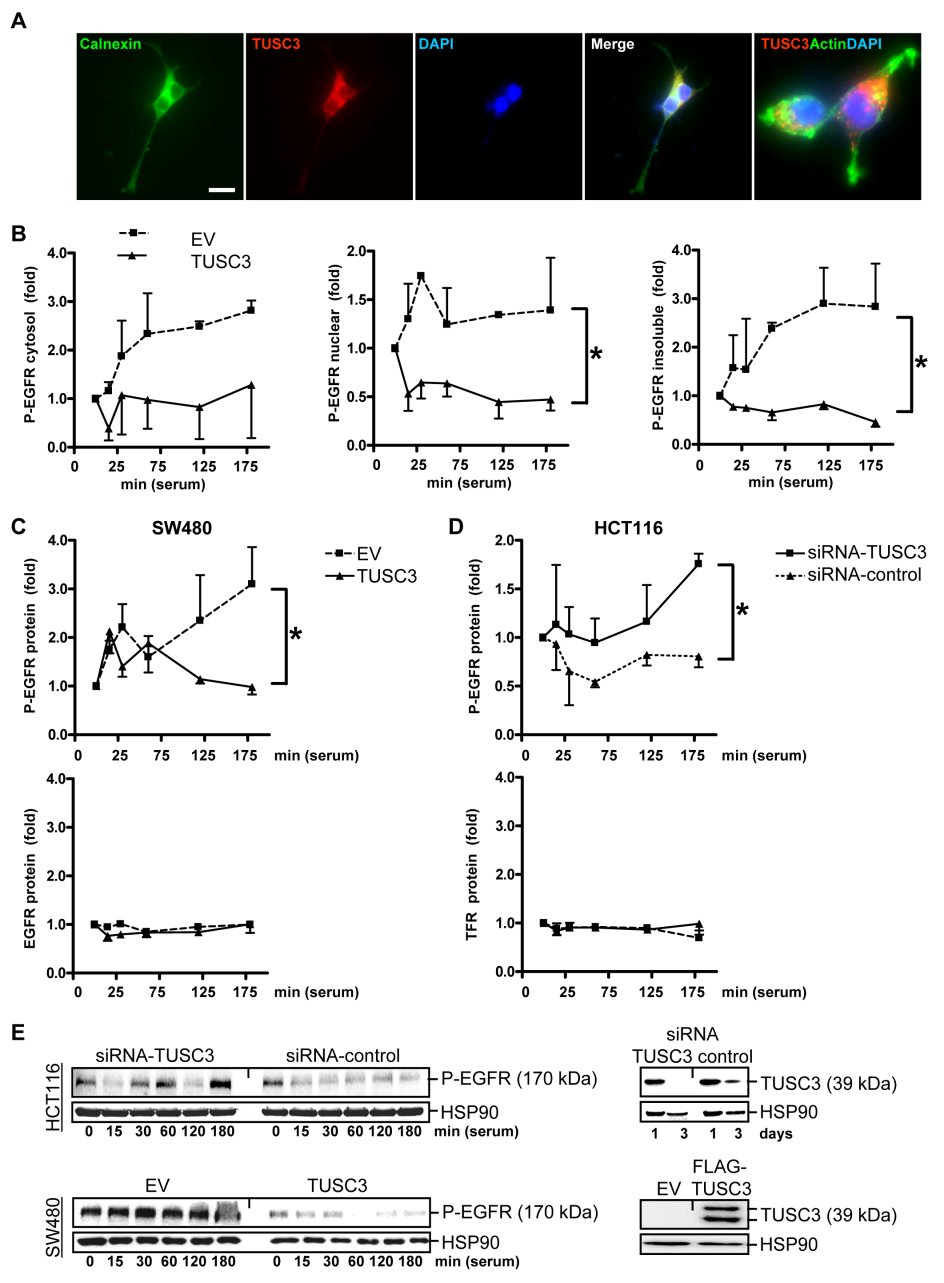
TUSC3 inhibits EGFR phosphorylation **A.**, Subcellular localization of ectopic TUSC3 protein at the ER. SW480 cells were transfected with FLAG-TUSC3 plasmid, fixed and stained for immunofluorescence microscopy. Color legend: red = FLAG-TUSC3, green = calnexin (ER marker) or phalloidin (actin), blue = nuclei (DAPI). Overlay of TUSC3 with calnexin or actin appears in yellow. Magnification 630x. **B.**, TUSC3 blocks tyrosine phosphorylation of EGFR/Her1. SW480 cells were transfected with TUSC3 and EV plasmids for 24 h, followed by serum removal (“starvation”) for 16 h and a restimulation with 20 % FCS (“serum shock”) for 0 to 3 h before cell harvest. Cells were then subjected to subcellular fractionation (SCF). Western blot analyses were done with an Ab against the C-terminal cytoplasmic (intracellular) domain containing phospho-tyrosine residue Y1068 important for EGFR activity (P-EGFR). O.D. values from bands in gels were calculated as -fold ± S.E. (*p < 0.05 TUSC3 *vs.* EV, Two-way ANOVA, *n* = 3). **C.**, TUSC3 inhibits EGFR phosphorylation without affecting total cellular EGFR protein levels. Cells were transfected and treated as in B and total cell lysate (TCL) was subjected to Western blot using the C-terminal Ab against the phosphorylated (Y1068) (*p < 0.05 TUSC3 *vs.* EV, Two-way ANOVA, *n* = 3) and unphosphorylated intracellular domain of the EGFR (n.s.). Data are calculated as in B. Similar results were obtained for the transferrin receptor (TFR/CD71). **D.**, TUSC3 knock-down increases EGFR phosphorylation. HCT116 cells were transfected with siRNAs and analyzed for P-EGFR in TCL as in B (*p < 0.05 TUSC3-siRNA *vs.* control-siRNA, Two-way ANOVA, *n* = 4). **E.**, Representative Western blots of the experiments in C-D are shown.

Since growth factor receptors need N-glycosylation for their function, we tested whether TUSC3, as a subunit of the ER-bound OST complex [14, 15], alters their activity in human CRC cells. EGFR was selected as one exemplary target of ER-mediated N-glycosylation. First, localization of ectopic TUSC3 protein to the ER was confirmed (Figure 2A), as shown previously for other cell types [14]. Accordingly, gene signatures of TUSC3-overexpressing SW480 cells identified “N-glycan biosynthesis” and “protein processing in endoplasmic reticulum” by gene set enrichment analysis (GSEA) (S5).

Next, we studied the impact of TUSC3 on phosphorylation, downstream signaling and subcellular localization of the EGFR. SW480 cells were transiently transfected with empty vector (EV) or TUSC3 expression plasmid, respectively, and underwent serum removal (“starvation”) for 16 h and subsequent restimulation with fetal calf serum (FCS) (“serum shock”) to trigger internalization, endosomal sorting, recycling or lysosomal degradation of the EGFR [16]. Cells were then subjected to subcellular fractionation (SCF) and Western blot analysis using an antibody (Ab) against the phosphorylated Y1068 residue in the intracellular C-terminal domain of the EGFR (Figure 2B). This approach evinced that the amount of phosphorylated EGFR (P-EGFR) was reduced by TUSC3. The decrease was observed in all three cell compartments: the insoluble (i.e. membrane / matrix / cytoskeleton) fraction (2.0±0.3 *vs*. 0.7±0.1) and the soluble extracts of the nucleoplasm (1.3±0.1 *vs*. 0.6±0.1) and the cytosol (2.0±0.3 *vs*. 0.9±0.1) (*p < 0.05 EV *vs.* TUSC3, Two-way ANOVA, *n* = 3). Similar results were obtained from total cell lysate (TCL) (Figure 2C), indicating that TUSC3 diminishes phosphorylation of the EGFR at the intracellular domain which is responsible for receptor dimerization and initiation of downstream signaling [16, 17].

Importantly, TUSC3 did not affect total EGFR protein levels (Figure 2C). When extracting TCL, no decrease of total EGFR protein was observed, suggesting that TUSC3 does not promote EGFR degradation. Similar results were obtained for the transferrin receptor (TFR/CD71) used as a control. *Vice versa*, RNAi knock-down of TUSC3 elevated EGFR phosphorylation (at Y1068) (Figure 2D). HCT116 cells with endogenous TUSC3 protein expression were transiently transfected with TUSC3-siRNA or control-siRNA for 48 h followed by starvation, serum shock and Western blot of TCL as detailed above (1.2±0.1 *vs*. 0.8±0.1, *p < 0.05 TUSC3-siRNA *vs.* control-siRNA, Two-way ANOVA, *n* = 4) (Figure 2E). The percentage of the EGFR at the cell surface was unchanged upon transfection with TUSC3, as demonstrated by quantitative flow cytometry (FC), using an Ab directed against the extracellular N-terminal domain of the EGFR (S6). Surface TFR/CD71 was not altered either. These data revealed that TUSC3 inhibits phosphorylation of the EGFR at the C-terminal domain responsible for dimerization and initiation of downstream signaling without altering its presence at the plasma membrane or inducing degradation.

**Figure 3:**
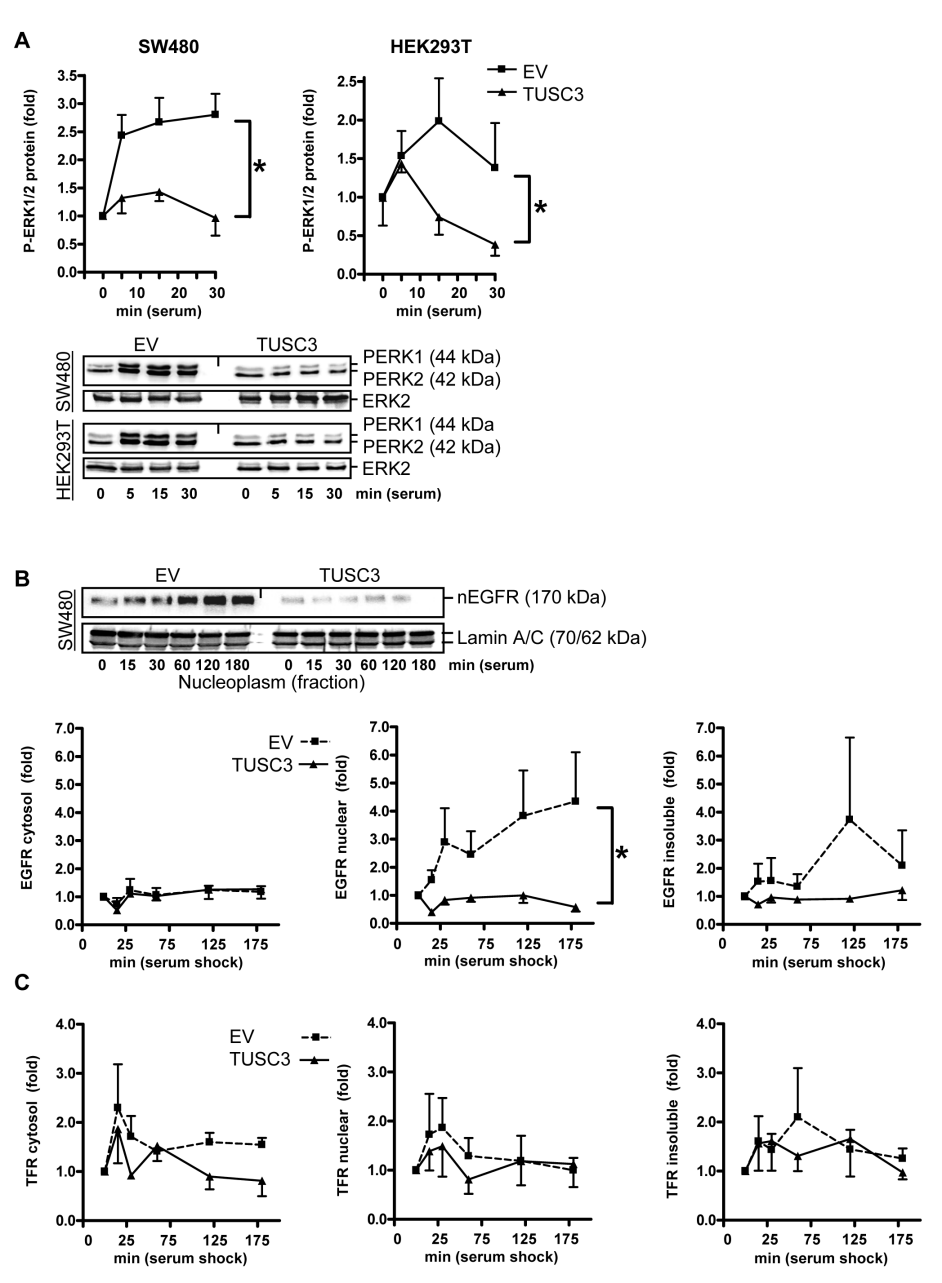
TUSC3 inhibits EGFR down-stream signaling and compartmentalization **A.**, TUSC3 reduces phosphorylation of ERK1/2. SW480 and HEK293T cells were transfected with TUSC3 or EV plasmids, starved and stimulated as described in Figure 2B. Quantitation and representative Western blots are shown. O.D. values from bands in gels were calculated as -fold ± S.E. (*p < 0.05 TUSC3 *vs.* EV, Two-way ANOVA, *n* = 3). **B.**-**C.**, TUSC3 reduces nuclear accumulation of the EGFR (B) but not of the control transferrin receptor (TFR/CD71) (C). SW480 cells were transfected with TUSC3 and EV plasmids for 24 h, followed by serum removal for 16 h and restimulation with 20 % FCS for 0 to 3 h before cell harvest to evoke endocytosis of the EGFR. Cells were then subjected to SCF, and Western blots were done with an Ab against the C-terminal cytoplasmic (intracellular) domain of the EGFR and quantified as in A (*p < 0.05 TUSC3 *vs.* EV, Two-way ANOVA, *n* = 3). Representative Western blots are shown above the bar graphs.

We then determined whether TUSC3 inhibits downstream signaling of the EGFR. SW480 and HEK293T cells were transfected with TUSC3 or EV plasmids, starved as above and then subjected to serum shock for 0 to 30 min. Western blot analysis of TCL revealed that TUSC3 prevented rapid serum-induced phosphorylation of ERK1/2, two kinases involved in cell proliferation and survival downstream of the EGFR (SW480: 2.2±0.4 *vs.* 1.2±0.1; HEK293T: 1.5±0.2 *vs.* 0.9±0.2, *p < 0.05 EV *vs.* TUSC3, Two-way ANOVA, *n* = 3) (Figure 3A). Conversely, RNAi knock-down of TUSC3 in HCT116 cells augmented ERK1/2 phosphorylation, whereas, neither overexpression nor knock-down of TUSC3 altered AKT phosphorylation (S7).

As genomic read-outs for the EGFR-RAS-ERK1/2 signaling cascade and to test the effect of TUSC3 on other downstream signaling pathways, luciferase reporter assays were conducted (S7). Interestingly, TUSC3 overexpression inhibited reporter gene activities dependent on hypoxia (0.6±0.2 *vs*. 0.4±0.1) and Wnt signaling (0.8±0.1 *vs*. 0.4±0.1) (*p < 0.05 EV *vs.* TUSC3, Two-way ANOVA, *n* = 3). Reciprocal results were obtained with siRNA.

Emerging evidence suggests that ErbB receptor family members undergo nuclear translocation to regulate transcription and promote cell survival or proliferation, a phenomenon which has been associated with poor prognosis in cancer patients [18, 19]. We therefore assessed whether TUSC3 alters subcellular distribution of the EGFR (Figure 3B). SW480 cells were transfected with TUSC3 or EV plasmids, followed by starvation and serum shock for 0 to 3 h and subsequent fractionation as described before. Western blot analyses showed that TUSC3 reduced the amount of EGFR in the nuclear fraction compared with the EV control (2.7±0.5 *vs*. 0.8±0.1, *p < 0.05 EV *vs.* TUSC3, Two-way ANOVA, *n* = 3). EGFR protein was not altered in the cytoplasmic and insoluble fractions. Moreover, TUSC3 had no effect on localization of the transferrin receptor (TFR/CD71) (Figure 3C), alluding at a potential specificity of TUSC3 towards the EGFR. However, future experiments have to clarify whether TUSC3 also affects other members of the ErbB family or related growth factor receptors. Taken together, these data indicated that TUSC3 inhibits EGFR phosphorylation and down-stream signaling in human CRC and non-cancer cells.

### TUSC3 confers resistance to the N-glycosylation inhibitor tunicamycin

**Figure 4:**
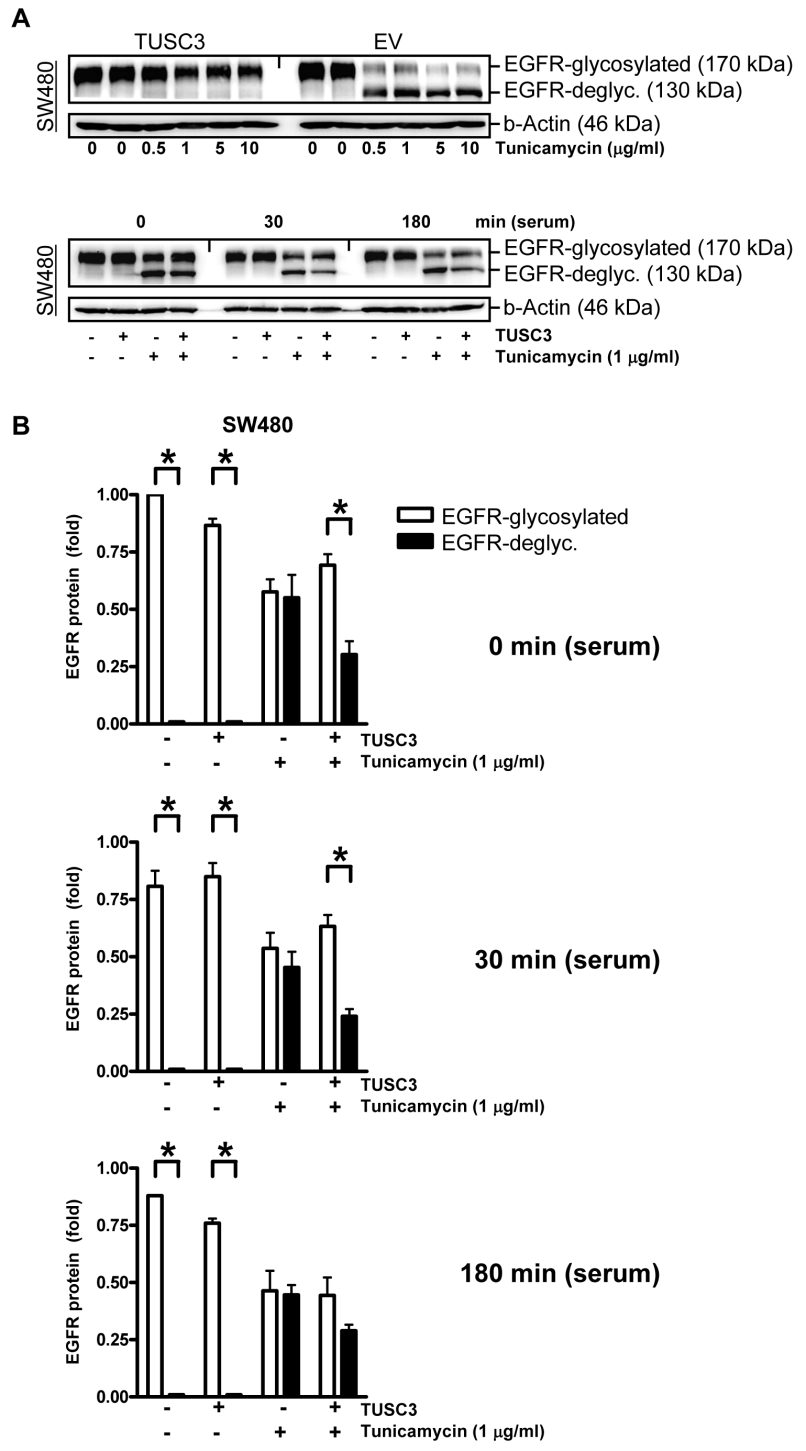
TUSC3 prevents tunicamycin-dependent EGFR deglycosylation **A.**, TUSC3 protects N-glycosylated EGFR in presence of the glycosylation inhibitor tunicamycin. Upper panel: SW480 cells were transfected with TUSC3 and EV plasmids for 6 h, followed by an incubation in presence or absence of tunicamycin (µg/ml) for additional 18 h. Lower panel: SW480 cells were transfected as above, followed by serum removal for 16 h in presence and absence of tunicamycin (1 µg/ml) and restimulation with serum (20 % FCS) for 0, 30 min to 3 h. TCLs were extracted for Western blotting using the C-terminal EGFR Ab. Representative gels from concentration- and time-dependent responses are shown. **B.**, Quantitative analyses of Western blots in A. The O.D. values from bands in gels are calculated as -fold ± S.E. (*p < 0.05 EGFRp170 *vs*. EGFRp130, Two-way ANOVA, *n* = 3).

To corroborate the link between TUSC3 and N-glycosylation of the EGFR, SW480 cells were transfected with TUSC3 or EV plasmids and incubated with tunicamycin, a pharmacological inhibitor of the first step of N-glycosylation upon translation across the ER membrane (Figure 4A) [20]. Western blot analyses of TCL using the C-terminal EGFR Ab demonstrated that two bands of 170 kDa and 130 kDa appeared upon tunicamycin treatment. The 130 kDa band has been described to correspond to the un-glycosylated form of the EGFR, while the 170 kDa band represents the fully glycosylated EGFR. Notably, TUSC3 overexpression (Figure 4B) or siRNA knock-down (S8) *per se* did not change the EGFR p170/p130 ratio. Instead, TUSC3 prevented tunicamycin-mediated deglycosylation of the EGFR (30 min: 0.7±0.1 *vs*. 0.2±0.1, *p < 0.05 EGFR p170 *vs*. p130, Two-way ANOVA, *n* = 3). Conversely, siRNA knock-down of TUSC3 in HCT116 cells increased tunicamycin-mediated deglycosylation of the EGFR (S8). In other words, TUSC3 was able to rescue the negative effect of tunicamycin on EGFR glycosylation, providing evidence that TUSC3 is sufficient but not essential for EGFR N-glycosylation.

### TUSC3 reduces cell viability and promotes apoptosis of human CRC cells

**Figure 5:**
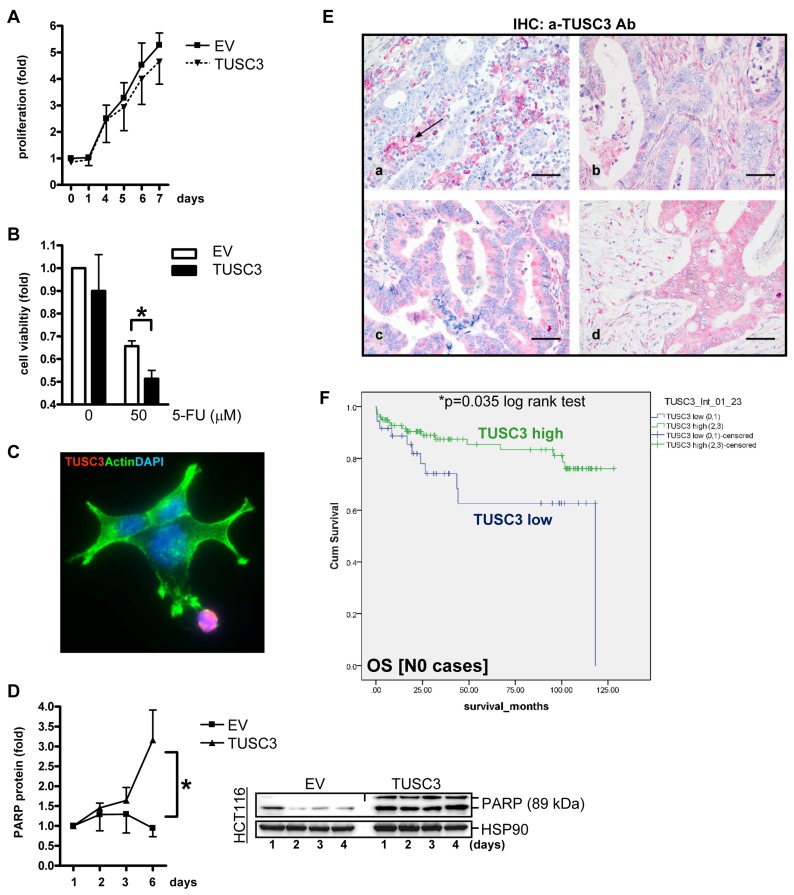
TUSC3 enhances CRC cell death and is associated with prognosis of CRC patients **A.**, Growth kinetics. SW480 cells were transfected with TUSC3 or EV plasmid, and proliferation was measured by colorimetric MTT assay after 1 to 7 days. O.D. values were calculated as -fold ± S.E. compared with day 0 (n.s., Two-way ANOVA, *n* = 3). **B.**, Chemoresistance response. SW480 were transfected for 24 h as in A, followed by an incubation with 5-FU (at 50 µM), and cell viability was determined after 48 h. Data were calculated as in A compared with vehicle control (*p < 0.05 EV *vs.* TUSC3, Two-way ANOVA, *n* = 3). **C.**-**D.**, TUSC3 promotes cell death (apoptosis). SW480 cells were transfected with TUSC3 or EV and incubated for 1 to 6 days. Color code: red = FLAG-TUSC3; green = actin (phalloidin); blue = nuclei (DAPI); magnification x630. Note the spread-out morphology of live TUSC3-negative cells in green *vs*. round dying TUSC3-positive cells in red. Representative immunofluorescence images (C) and quantitative Western blot analyses (D) from TCL (all 3 cell lines) are shown which detect cleaved PARP (apoptosis marker). Data are -fold ± S.E (*p < 0.05 EV *vs.* TUSC3, Two-way ANOVA, *n* = 3). **E.**, TUSC3 is lost in a subset of CRCs. Tissue microarrays (TMAs) with tumor specimens from CRC patients (*n* = 306) were stained by immunohistochemistry (IHC). Representative images: (a) TUSC3 negative tumor cells [score 0] with black arrow marking TUSC3+ blood-derived plasma cells (positive control); (b-d) TUSC3+ tumor cells [(b) score 1, (c) score 2, (d) score 3] and stroma. **F.**, Kaplan-Meier-Survival-Analysis. TUSC3 protein expression and its correlation to prognosis (Supplementary Table S1-S3) and clinical factors (Table S4) was calculated. Combined scores for the intensity and frequency of TUSC3 staining in tumor and stroma tissue are expressed as: 0 = negative (0-25 %), 1+ = weak positive (25-50%), 2+ = moderate positive (50-75 %), 3+ = strong positive (75-100 %). Node negative (N0) patients with low (*scores 0-1*) TUSC3 expression have a reduced overall survival (OS) probability compared with patients showing high (*scores 2-3*) TUSC3 expression (*n* = 135, *p = 0.035, log rank test).

To assess the effect of TUSC3 on cell viability, colorimetric MTT assays were conducted in human CRC cell lines. For growth kinetics (Figure 5A), SW480 cells were transfected with TUSC3 or EV plasmids, respectively, and proliferation was measured after 1 to 7 days. TUSC3 did not lower the proliferation rate compared with the EV control. To test, whether TUSC3 alters the cellular sensitivity to chemotherapeutics (Figure 5B), SW480 were transfected as above for 24 h, followed by an incubation with 5-fluorouracil (5-FU, at 50 µM), and cell viability was determined after 48 h. TUSC3 diminished cell survival compared with the EV control (0.7±0.02 *vs*. 0.5±0.04, *p < 0.05 EV *vs.* TUSC3, *t*-test, *n* = 3). However, TUSC3 reduced cell viability also in the absence of the drug (dose 0). We therefore asked whether TUSC3 promotes cell death by apoptosis. SW480 were transfected with TUSC3 or EV plasmids for 24 h, followed by fixation and staining for immunofluorescence microscopy (Figure 5C). Cell death was observed in TUSC3-transfected cells with rounded-up cells, membrane “blebs” and fragmentation of the nuclei compared to EV control cells with an adherent “spread-out” epithelial morphology and cytoskeleton. Western blot analysis using TCL from SW480, HCT116 and HEK293T cells transfected for up to 4 days demonstrated that TUSC3 increased the amount of the cleaved pro-apoptotic protein PARP (3-fold, *p < 0.05 EV *vs.* TUSC3, Two-way ANOVA, *n* = 3) (Figure 5D) and caspase-3 (not shown). Thus, taken together, TUSC3 reduces cell viability and promotes apoptosis in human CRC cells.

### Loss of TUSC3 protein in CRC correlates with poor survival of CRC patients

Since we had shown that TUSC3 is frequently lost in early carcinogenesis and its absence correlates with enhanced cell survival, we aimed to assess the role of TUSC3 loss in the clinical course of CRC patients. We conducted an immunohistochemical analysis in a large cohort of CRC patients (*n* = 306 cases) (Supplementary Table S1). Analysis of the stainings evinced a loss of TUSC3 positivity in approx. 30% of cases, similar to *TUSC3* promoter methylation and mRNA down-regulation, confirming the existence of a subset of TUSC3-negative tumors. Of note, TUSC3 was localized to mononuclear blood cells as a positive control, but was also found in the tumor and stroma (non-epithelial) compartments of CRC tissue (Figure 5E). Scores for frequency and intensity of TUSC3 positivity were correlated to overall survival (OS) in Kaplan-Meier-plots (Figure 5F). Patients with CRC without nodal involvement (N0) showed low TUSC3 protein expression (*scores 0-1*) and had a significantly worse prognosis than those with increased TUSC3 protein expression (*scores 2-3*) [OS: 82.6 ± 9.7 (*n* = 36) *vs.* 108.4 ± 4.6 (*n* = 99) months, *p = 0.035, log rank test].

Interestingly, this observation was only evident in node negative patients (Supplementary Table S2), whereas in patients with lymph node metastasis (N+) no association with prognosis was stated (Supplementary Table S3). Combined analysis grouped by nodal status (N) and tumor stage (T) confirmed a trend for survival benefit of TUSC3-positive patients (T2N0) [OS: 58.7 ± 14.6 (*n* = 8) *vs.* 109.8 ± 7.7 (*n* = 32) months, *p* = 0.080, log rank test], whereas this prognostic advantage disappeared upon tumor progression (T3/4N0) (data not shown). No associations were recorded for other clinical variables including patient characteristics (age, gender) and tumor parameters (Supplementary Table S4). The frequent loss of TUSC3 protein already in adenomas (S4), together with its prognostic implication for patients with early CRC without lymph node metastasis, again pointed to an important role of TUSC3 in early events of tumorigenesis. Supportive analysis of an independent cohort of CRC patients using cBioPortal of Cancer Genomics [Colorectal Adenocarcinoma, TCGA, Provisional, *n* = 633] (Supplementary Table S5 and S9) evinced that *TUSC3* gene alterations, mainly deletions and missense mutations, conferred poor clinical outcome when combined with those in ErbB receptors, indicative of a possible cooperation of these pathways as proposed from our experiments in CRC cell lines. Similar results were obtained from breast cancer patients, a tumor entity where gene alterations in ErbB receptors (e.g. amplifications, mutations) are common and targets for clinical therapy. Spearman correlation plots detected an inverse correlation of *TUSC3* methylation and mRNA expression in two data sets of colorectal adenocarcinoma (S10) [TCGA, Provisional (*n* = 633 cases); TCGA, Nature 2012 (*n* = 195 cases)]. TUSC3 methylation was also higher in cases with microsatellite instability (MSI-H) and methylator phenotype (CIMP-H) compared with the respective controls, emphasizing that TUSC3 silencing may correlate with certain molecular subtypes of CRC. However, TUSC3 methylation *per se* did not contribute to patient survival (S10), confirming our findings that TUSC3 silencing occurs already in benign adenomas and on the protein level predicts prognosis only at early stage CRC but not in advanced mCRC.

## DISCUSSION

In this study, we characterized the clinical relevance of tumor suppressor candidate 3 (TUSC3) in CRC. Our data show that (i) the *TUSC3* gene was silenced by DNA methylation of its promoter in tumor tissue of a large subset of CRC patients, (ii) loss of *TUSC3* mRNA expression by DNA methylation was an early event already in adenomas and also detectable by reduced TUSC3 protein levels in resected benign adenomatous polyps, and (iii) low TUSC3 protein positivity in early stage CRC patients correlated with poor clinical outcome. As an exemplary target of N-glycosylation, where TUSC3 constitutes one subunit of the OST multiprotein complex at the ER membrane [14, 15], we selected EGFR/Her1, a growth factor receptor targeted by therapeutic drugs and Abs (e.g. cetuximab). We demonstrate that TUSC3 reduced tumor cell viability and enhanced apoptosis, an effect which may be in part attributed to the ability of TUSC3 to inhibit phosphorylation and down-stream signaling of the EGFR together with additional oncogenic pathways including ERK1/2, hypoxia and Wnt signaling. Since other ErbB family members and a plethora of membrane and secretory proteins are all subjected to OST-mediated N-glycosylation [7-11], our data pin-point only one possible candidate affected by TUSC3 in CRC, and many more OST substrates are likely to be altered upon TUSC3 loss. Thereby, we may provide a more general link between enzymes that regulate protein glycosylation and cancer [7]. The main effector pathway of TUSC3 previously shown in prostate, ovarian and pancreatic cancer cells comprised the control of the ER stress response *via* modulation of MGAT enzymes and BCL family proteins, involved in cell survival and regulation of apoptosis [21-23]. This pattern was confirmed in our study in CRC cells. Importantly, in addition to this established pathway, our data provide a possible mechanism of post-translation regulation of membrane receptors by TUSC3 in CRC, exemplified by EGFR/Her1.

We found that TUSC3 prevented tunicamycin-mediated deglycosylation of the EGFR [20]. Thus, TUSC3 rescued the negative effect of tunicamycin on EGFR N-glycosylation, proposing that TUSC3 positively contributes to EGFR N-glycosylation in the ER. Notably, loss of TUSC3 did not prevent N-glycosylation *per se*, proposing a non-essential or at least redundant role of TUSC3 in this process catalyzed by the OST-complex which can override and compensate for the absence of TUSC3, at least in case of the exemplary target protein studied here. TUSC3 is a subunit of an enzyme that acts on a third of the proteome, hence, attributing its effects to a single target seems unlikely [14, 15]. Since N-glycosylation is critical to the proper folding of the overwhelming majority of membrane and secreted proteins, it is most probable that loss of TUSC3 in tumors has pleiotropic effects that act additively to promote tumor growth.

One major finding of the present study was that TUSC3 enhanced intracellular accumulation of the EGFR without changing its presence at the cell surface. Ample evidence [17-19] describes the retrograde transport of ErbB receptors from the ER-Golgi system back to the nuclear envelope which is continuous with the perinuclear ER-membrane [24-27] and subsequent translocation into the nucleus [28, 29]. Intracellular ErbB fragments can also be generated by proteolytic cleavage [18, 19]. Thereby, ErbB receptors are thought to interact with transcription factors (e.g. STATs) which drive promoters of pro-oncogenic genes (e.g.*CCND1, MYC, NOS2, BCLXL, MMP2*) [30-33]. Furthermore, EGFR recruits chromatin modifiers (e.g. HDACs, SRCs) [31, 34] and inhibits p53-mediated apoptosis [35]. Importantly, nuclear localization of ErbB receptors is a negative predictor for survival of cancer patients [18, 30]. Nuclear EGFR has also been connected to cellular resistance to radiation and targeted therapies including cetuximab or gefitinib [36-38]. Thus, in absence of TUSC3, EGFR may be defective in guided membrane trafficking and more prone for misallocation within the cell.

**Figure 6:**
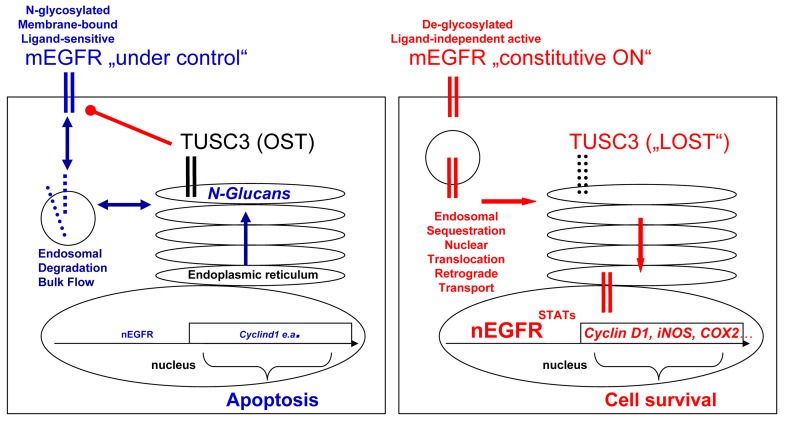
Model for TUSC3 functions in CRC Left panel: In normal cells, TUSC3 is a part of the OST multiprotein complex in the ER which contributes to correct N-glycosylation, folding, transport and insertion of proteins (e.g. the EGFR) into or, in case of secreted factors, release from the plasma membrane. N-glycosylation also participates in the control of ligand-sensitivity, plasma membrane retention and subsequent internalization, sorting and degradation of growth factor receptors, in order to limit and terminate their signaling. Right panel: In cancer cells, loss of TUSC3 alters OST-mediated processes causing defects in posttranslational modification, processing and traffic of membrane (and secretory) proteins. For example, aberrant retention times of the EGFR at the plasma membrane and/or intracellular sequestration of the receptor from extracellular ligands enables constitutive signaling. Retrograde transport of the EGFR from the perinuclear Golgi-ER network to the nuclear envelope (or proteolytic cleavage) may facilitate accumulation of EGFR in the nucleus, where it can act independently of ligand and activate transcription of proto-oncogenes at the DNA. Legend: mEGFR = cell surface EGFR, nEGFR = nuclear EGFR, Blue = tumor suppressive (response) signaling; red = tumor promoting (resistance) signaling.

From the existing literature, it is yet unclear whether N-glycosylation renders the EGFR more or less active [22]. Since we show that TUSC3 inhibits EGFR signaling, our work may contribute to answer this question. Taken together, we propose the following model (Figure 6): in non-malignant cells, TUSC3 is a part of the OST multiprotein complex in the ER which promotes N-glycosylation of EGFR, one out of many exemplary targets, and facilitates its processing, folding, transport and insertion [22] into the plasma membrane [7, 23] where it is ready for ligand binding, dimerization and signal initiation. N-glycosylation is also a determinant for timely plasma membrane retention of the EGFR, which enables internalization (endocytosis), endo-lysosomal sorting and degradation of the receptor in order to limit and terminate signaling [16, 24]. Upon silencing of TUSC3 in cancer cells, this fine-tuned regulation at the plasma membrane is impaired. Altered retention times and spatial sequestration of EGFR from its extracellular ligands or intracellular recycling and degradation routes may facilitate constitutive EGFR signaling. Moreover, nuclear EGFR may act independently of ligand to activate transcription of proto-oncogenes. Further studies have to explore the spatial compartmentalization of EGFR and other growth factor receptors to elucidate the role of these mechanisms for resistance to EGFR-targeted therapies.

In conclusion, our study revealed that TUSC3 is down-regulated early in CRC development by epigenetic silencing. In human CRC cells, absence of TUSC3 increased cell survival and prevented apoptosis. In this context, the EGFR may be one of many ER-bound protein substrates suffering from aberrant regulation upon TUSC3 loss. Our data thereby reveal a potential link between post-translational modification and carcinogenesis. Accordingly, loss of TUSC3 was associated with unfavorable outcome in early stage CRC, indicating that this subset of patients may require intensified monitoring and may benefit from additional adjuvant therapies.

## MATERIALS AND METHODS

### Subjects

Specimens were obtained from CRC patients undergoing surgery at the university hospitals in Munich, Kiel and Mannheim. Tissues were obtained during resection of the primary tumors or as biopsies and were either snap-frozen in liquid N2 or formalin-fixed and paraffin-embedded (FFPE). Histology was verified by an expert pathologist (CR, Kiel). Informed consent was obtained from the patients prior to enrolment in the study. The study was approved by the ethics committees of the Universities of Munich, Kiel and Heidelberg.

### Reagents

Chemicals were from Merck (Darmstadt, Germany) and Sigma (Steinheim, Germany). Antibodies were FLAG (#F7425, #1804, Sigma), phospho-ERK1/2 (p44/p42) (#4370), ERK1/2 (#4695), phospho-AKT (#2965, #4058), pan-AKT (#4691), AKT2 (#3063), EGFR (recognizes the C-terminal intracellular domain, #4267), phospho-EGFR (recognizes the C-terminal intracellular domain, #3777) (all from Cell Signaling, Danvers, MA), transferrin receptor (CD71, sc-32272), HSP90 (sc-7947), lamin A/C (sc-20681), EGFR (recognizes the N-terminal extracellular domain, sc-120) (all from Santa Cruz, CA), TUSC3 (SAB4503183, Sigma), TUSC3 (ab77600, Abcam, Cambridge, UK) and TUSC3 (NBP1-55630, Novus, Littleton, CO). Pharmacologicals were tunicamycin, 5-fluorouracil (5-FU) and 5-aza-(2-deoxy)-cytidine (AZA) (all from Sigma).

### Primer design

Primers were designed with NCBI Primer Blast (http://www.ncbi.nlm.nih.gov/tools/primer-blast/), based on Primer 3 Software (Whitehead Institute for Biomedical Research, Cambridge, MA, USA), or provided by Epigenomics AG, Berlin, Germany. Primer sequences are listed in Supplementary Table S6.

### Nucleic acid isolation and bisulfite conversion

Nucleic acids were recovered from cell lines or frozen tissue samples using RNeasy Total RNA Mini or QIAmp DNA Mini Kits as recommended by the manufacturer (Qiagen, Hilden, Germany). Tissue sections were extracted according to instructions from the QIAamp DNA FFPE Tissue Kit (Qiagen). Bisulfite treatment was conducted on genomic DNA according to the manufacturer’s protocol (EpiTect Bisulfite Kit, Qiagen).

### Reverse transcription polymerase chain reaction (RT-PCR)

Reverse transcription of mRNA and PCR was conducted as described previously [39]. Primers are listed in Supplementary Table S6.

### MethyLight (ML) PCR

Methylation-specific quantitative real-time PCR was performed by MethyLight technology as detailed by the manufacturer (Qiagen) on a LightCycler 480 device with 1.5 LC480 software (Roche Diagnostics GmbH, Mannheim, Germany) [39]. The fluorescent probe was labelled with 3´ BHQ1 (black hole quencher one) and 5’ FAM (6-carboxyfluorescein reporter) which emits light at 520 nm similar to SYBR Green I. MethyLight conditions were: 10 min at 95°C for activation and 50 × cycles with 15 sec at 95°C, 30 sec at 60°C and 10 sec at 72°C (data acquisition step). PCR products were quantified by comparison to a standard curve (10-20 ng) of serial dilutions (1:0, 1:4, 1:16, 1:64, 1:256) of a fully methylated standard DNA (Qiagen). The ratio of methylated DNA (in ng) from the gene of interest (GOI) and the unmethylated reference gene (ACTB) was divided by that of the fully methylated standard DNA (STD) multiplied by factor 100 to yield the “percent methylation ratio” (PMR): PMR = [(QueryGOI/QueryACTB) / (STDGOI/STDACTB)]*100.

### Methylation-sensitive high resolution melting (HRM) analysis and next generation sequencing (NGS)

Genomic bisulfite-converted DNA samples were analyzed by methylation-sensitive high-resolution melting (HRM) as detailed previously [39]. Next generation sequencing (NGS) of PCR-products from bisulfite-converted genomic DNA was performed by means of MiSeq ultra-deep sequencing [40].

### DNA constructs

Full length (FL) TUSC3 (NM_006765) cDNA was PCR-amplified from total RNA of SW480 cells using GoTaq Green Mastermix (Promega GmbH, Neckarau, Germany) and inserted with and without N-terminal FLAG tag in pTARGET (pT) vector (Promega). Luciferase reporter plasmids HRE-luc, PPRE-luc, SRE-luc and TOPFLASH-luc were described elsewhere [41]. TUSC3-siRNA and control-siRNA oligonucleotides were from Dharmacon (SMARTpool: ON-TARGETplus, Thermo Scientific, Lafayette, CO).

### Cell culture and assays

Human cell lines were obtained from the Deutsche Sammlung von Mikroorganismen und Zellkulturen GmbH (DSMZ, Braunschweig, Germany) and American Type Culture Collection (ATCC, Rockville, MD and LGC Standards, Wesel, Germany) and cultured as suggested by the distributors. Transient transfection was done with Turbofect (Thermo Fisher Scientific, Waltham, MA). Colorimetric 3- [4,5-dimethyl thiazol-2-yl]-2,5-diphenyl tetrazolium bromide (MTT) assays were performed as published [41]. Flow cytometry (FC) was conducted on a FACSCanto II device (Becton Dickinson, Heidelberg, Germany) with analysis software (FACSDiva, Becton Dickinson). Immunofluorescence microscopy, preparation of total cell lysate (TCL), subcellular fractionation (SCF) and Western blotting were done as published [41].

### cDNA microarray and DNA mutation analysis

Total RNA was extracted from SW480 cells transiently transfected with TUSC3 or EV plasmids in triplicates, and cRNA was hybridized to two sets of microarrays (Affymetrix Gene ST 1.0). Gene set enrichment analysis (GSEA) was performed as described [41]. Ranplex CRC arrays were purchased from Randox Laboratories GmbH (Wülfrath, Germany), and genomic DNA hybridized and mutations analyzed as recommended by the manufacturer.

### Immunohistochemistry (IHC)

TUSC3 Ab (from Novus) was diluted 1:100 and staining was conducted as suggested by the manufacturer (Vectorlabs, Burlingame, CA). 3,3’-diamino benzidine DAB (brown color) or Fast Red Naphtol (red color) were used for detection. Staining positivity was determined in epithelial cells (tumor and normal colon) and stroma cells (lamina propria). Frequency and intensity of TUSC3 staining was evinced in custom-made (from CR) tissue microarrays (TMA) as detailed before [41]: Scores were: 0+ = negative (0-25% positive), 1+ = weak (25-50%), 2+ = moderate (50-75%), 3+ = strong (75-100%). H-scores were calculated according to the formula [1 × (% cells 1+) + 2 × (% cells 2+) + 3 x(% cells 3+)]. For Kaplan-Meier plots, cases were subjected to dichotome analysis (scores 0-1 = negative; scores 2-3 = positive).

### Statistics

Bioinformatic data were retrieved from cbioportal.org in accordance with the TCGA publication guidelines [42, 43]. Receiver operating characteristic (ROC) analysis was performed to optimize the PMR cutoff value which yields the best discrimination between non-neoplastic mucosa, adenoma and tumor samples [39]. PMR values > 30% were considered as methylated, whereas PMR levels < 30% were classified as unmethylated. Statistical calculations were done with the software Prism 4.0 (GraphPad Software, Inc., La Jolla, USA). IHC stainings from patient tissues were analyzed using SPSS version 20.0 (IBM Corporation, Armonk, NY). All tests were unpaired and two-sided, and *p*-values < 0.05 were considered significant (*).

## SUPPLEMENTARY MATERIALS FIGURES AND TABLES





## Abbreviations

Ab: antibody
ACTB: beta-actin
AD: adenoma
AKT: protein kinase B
AZA: 5-aza-(2-deoxy)-cytidine
BSP: bisulfite sequencing PCR
(m) CRC: (metastatic) colorectal cancer
DAB: 3,3’-diamino benzidine
EGFR: epidermal growth factor receptor
ER: endoplasmic reticulum
ERBB: Erb receptor tyrosine kinase
ERK: extracellular signal-regulated protein kinase
EV: empty vector
FC: flow cytometry
FCS: fetal calf serum
FFPE: formalin-fixed and paraffin-embedded
FITC: fluorescein isothiocyanate
FL: full length
5-FU: 5-fluorouracil
G: tumor grade
GOI: gene of interest
GSEA: gene set enrichment analysis
HDAC: histone deacetylase
HER: human EGFR
HRE: hypoxia response element
HRM: high resolution melting
NGS: next generation sequencing
NC: normal colon (tissue)
NT: normal/non-tumor
ML-PCR: MethyLight PCR
MSP: methylation-specific PCR
MTT: 3-(4,5-dimethylthiazol-2-yl)-2,5-diphenyltetrazolium bromide
O.D: optical density
OST: oligosaccharyltransferase
PARP: poly ADP-ribose polymerase
PMR: percent methylation ratio
PPRE: peroxisome proliferator-activated receptor responsive element
pTNM: primary tumor classification system
RAS: rat sarcoma viral oncogene homolog
ROC: receiver operating characteristic
RTK: receptor tyrosine kinase
SCF: subcellular fractionation
STD: fully methylated standard DNA
SRC: steroid receptor coactivator
SRE: serum response element
STAT: signal transducer and activator of transcription
STD: standard
TCL: total cell lysate
TFR: (CD71) transferrin receptor
TMA: tissue microarray
TOPFLASH: beta-catenin/Tcf4 responsive element
TU: tumor tissue
TUSC3: tumor suppressor candidate-3
