# Supplementary material for: Epigenetic silencing of tumor suppressor candidate 3 confers adverse prognosis in early colorectal cancer
Source: Oncotarget. 2017 Sep 15;8(49):84714–28. doi: 10.18632/oncotarget.20950 (PMC5689568; doi:10.18632/oncotarget.20950)
Supplement: Supplementary file 1 [file oncotarget-08-84714-s001.pdf]

Epigenetic silencing of tumor suppressor candidate 3 confers adverse prognosis in early colorectal cancer

SUPPLEMENTARY MATERIALS

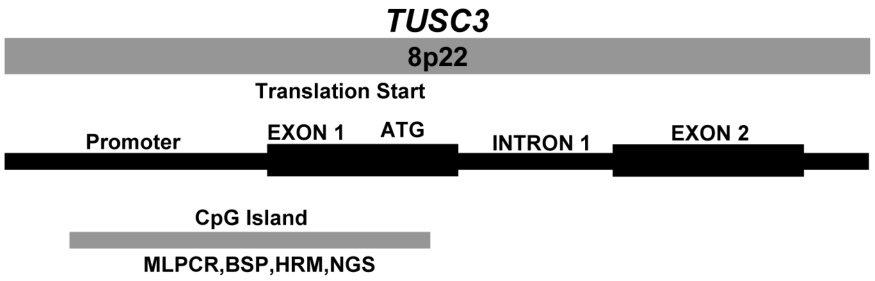

Supplementary Figure 1: Scheme of the human TUSC3 chromosomal locus. Organization of the proximal promoter and intron-exon structure of the human *TUSC3* gene and localization of CpG islands for detection by primers and fluorescence probes (as listed in Table S6).

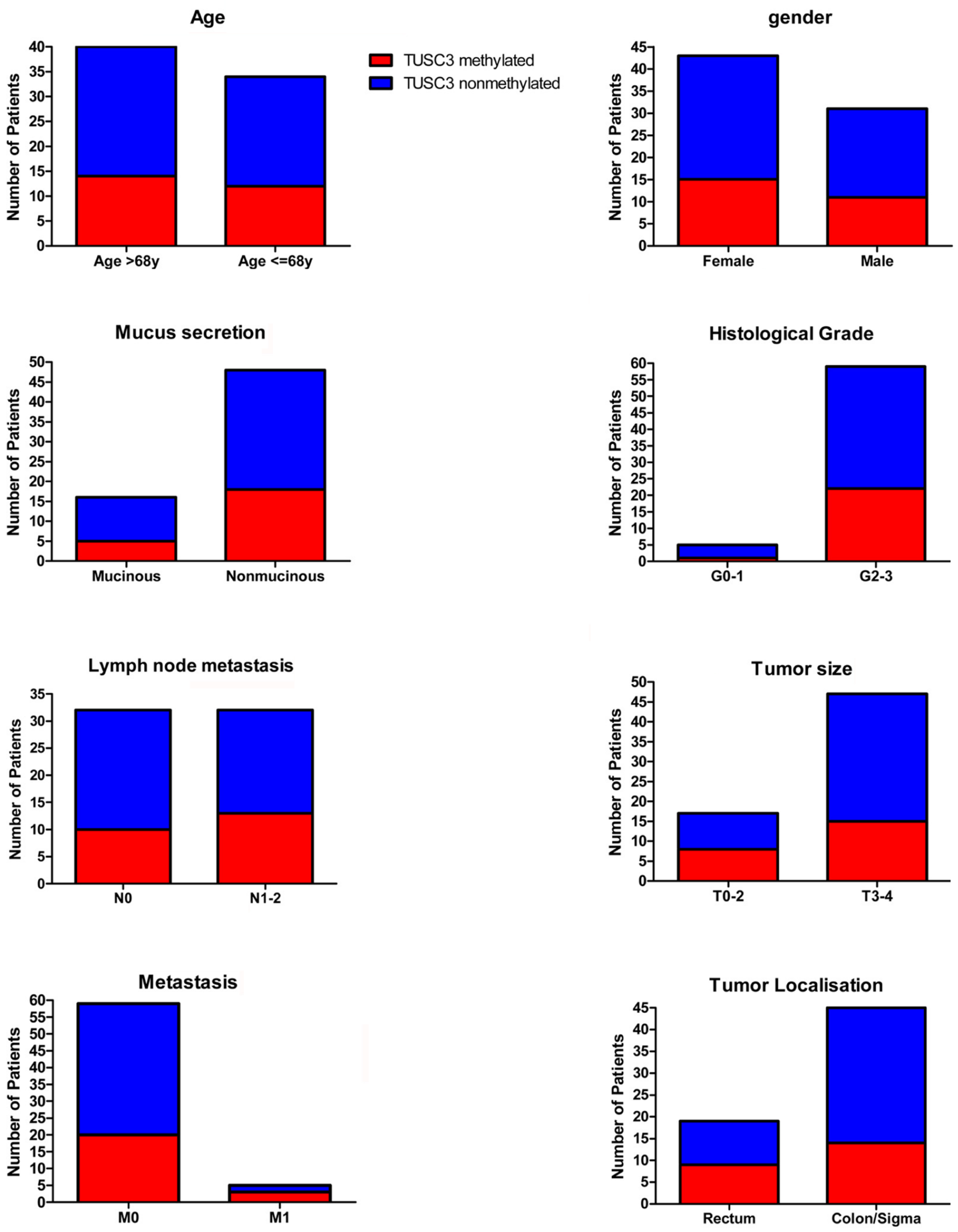

Supplementary Figure 2 Association of TUSC3 methylation with clinical factors in CRC patients. *TUSC3* methylation did not correlate with clinical characteristics. Absolute case numbers are shown per group (n.s., Fisher exact test, n = 64 cases). Legend: Red bars = TUSC3 methylated; Blue bars = TUSC3 non-methylated.

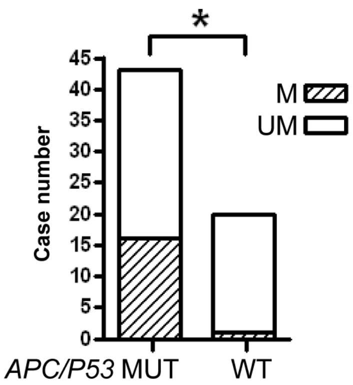

Supplementary Figure 3: Association of TUSC3 methylation with APC/P53 mutations in CRC patients. *TUSC3* methylation was correlated with *APC/P53* but not with *BRAF* or *KRAS* gene mutations in tumor samples using RanPlex CRC arrays (*p=0.0068, Fisher exact test, n = 63 cases). Legend: M = *TUSC3* methylated; UM = *TUSC3* unmethylated.

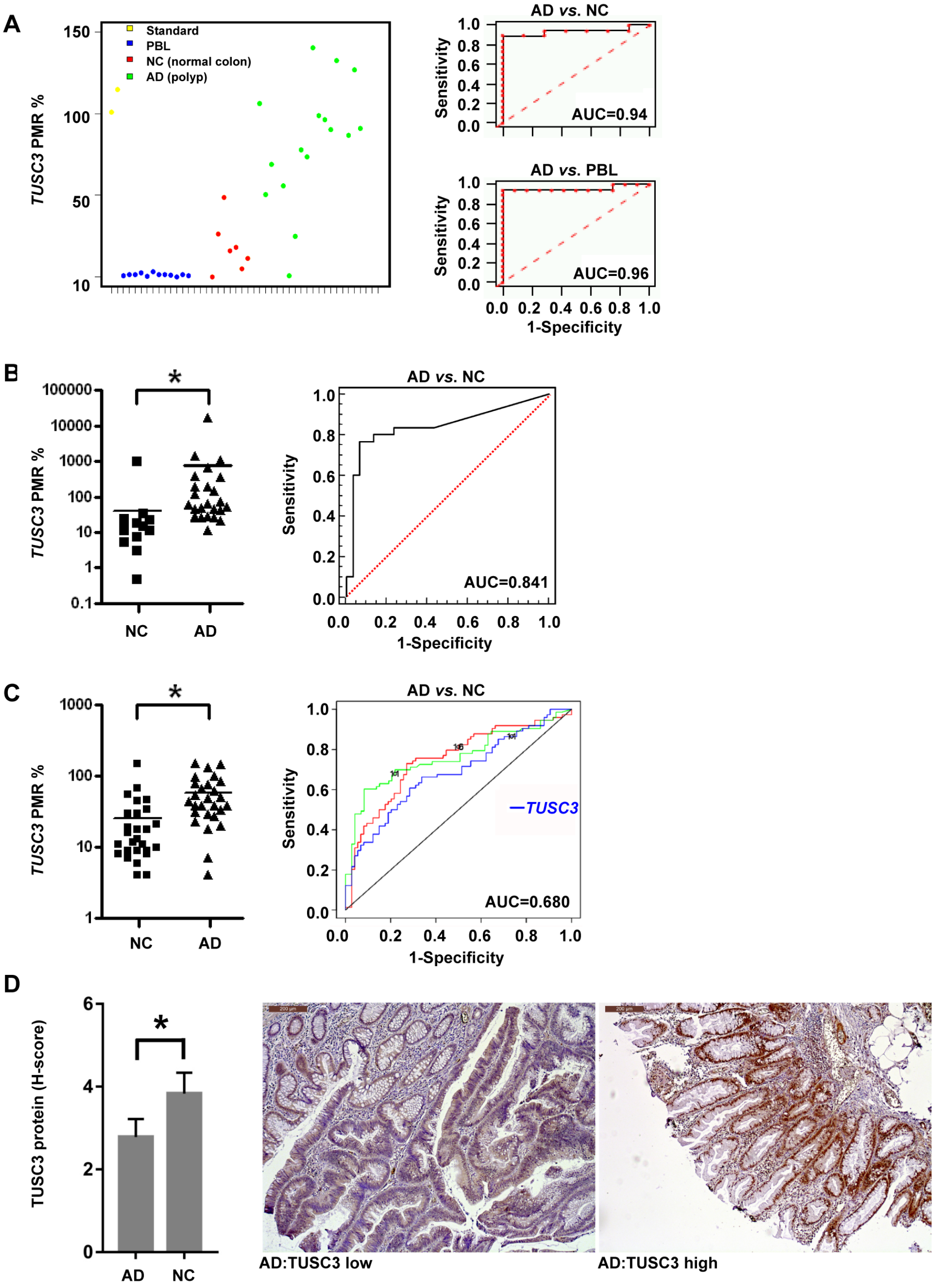

Supplementary Figure 4: TUSC3 methylation in adenomas and normal colon tissue. A, Detection of *TUSC3* promoter methylation in resected benign adenomatous polyps by ML-PCR. DNA was extracted from adenomas (AD, n=16 cases) and compared to normal colon (NC, n=7 cases) tissue and peripheral blood lymphocytes (PBL, n=12 cases) from healthy donors. Receiver Operating Characteristic (ROC) with "area under the curve" (AUC) values and PMR plots are shown. Sensitivity and specificity were maximized by choosing the optimal cut point according to the maximal Youden index J. B-C, Detection of *TUSC3* promoter methylation in (B) serum and (C) tissue samples from matched adenomas (AD) and normal colon (NC) by ML-PCR. PMR values are shown as in A (B: *p=0.0002, n=30 cases; C: *p=0.0001, n=27 cases; Wilcoxon signed rank test). D, Detection of TUSC3 protein in adenomas. FFPE tissue sections from adenomas (AD, n=16 cases) and adjacent normal colon (NC, n=16 cases) were stained by immunohistochemistry (IHC). H-scores were calculated for TUSC3 protein positivity and presented as mean ± S.E. (*p=0.0326, paired t-test). Representative images are shown.

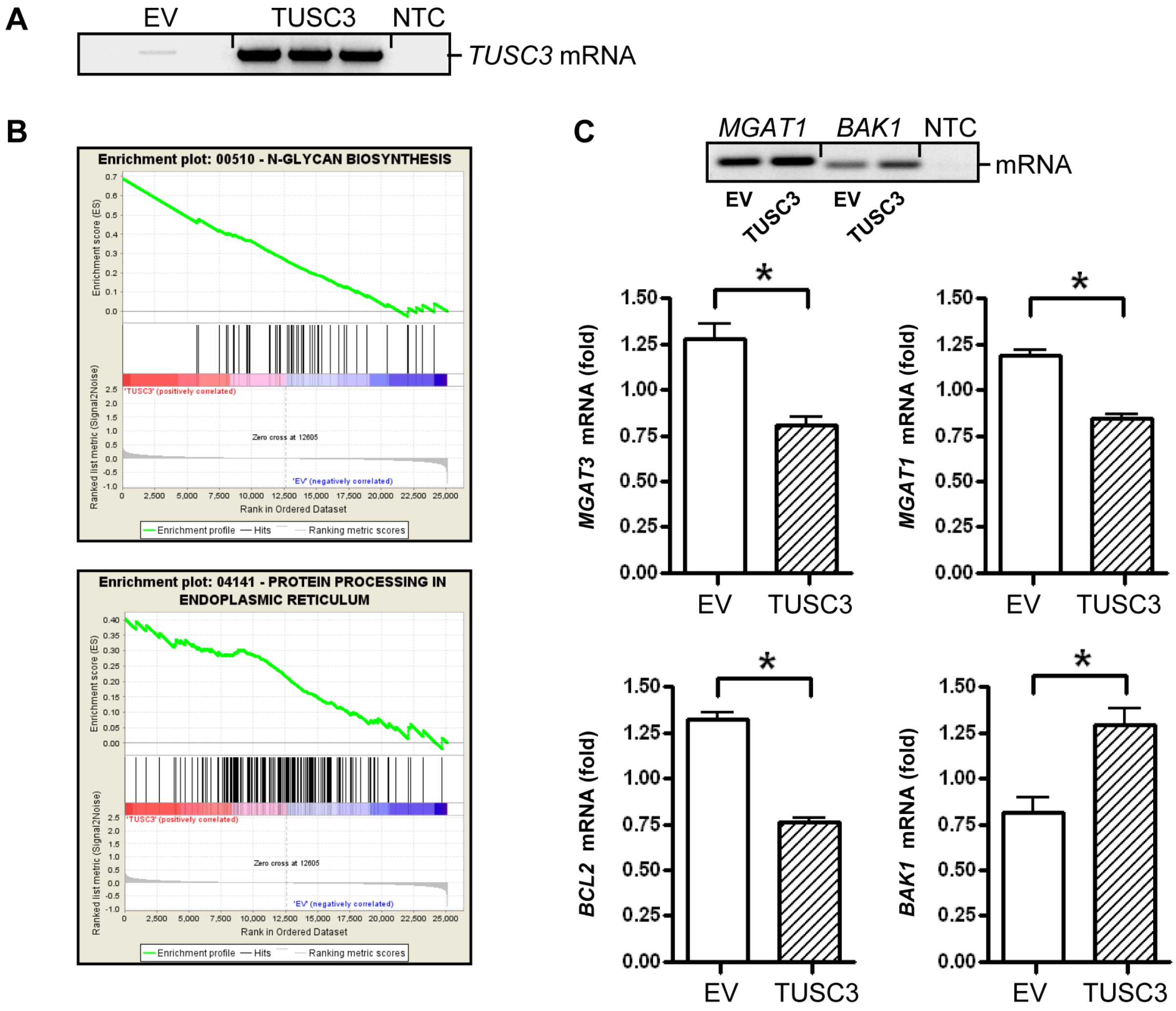

Supplementary Figure 5: TUSC3 regulates ER-associated gene expression. A, SW480 cells were transfected in triplicates with TUSC3 or EV plasmids for 24 h before total RNA extraction and hybridization of cRNA to cDNAmicroarrays. B, Gene signatures in TUSC3-expressing SW480 cells were identified by GSEA. Enrichment plots and heat-maps of two gene sets, N-GLYCAN BIOSYNTHESIS and PROTEIN PROCESSING IN ENDOPLASMIC RETICULUM with significance (*p<0.05 TUSC3 *vs.* EV, n=3 per plasmid). Color code: Red = enriched by TUSC3; Blue = enriched by EV. C, Validation of TUSC3-regulated genes (*MGAT1, MGAT3, BAK1, BCL2*). RT-qPCR analyses are shown next to representative agarose gels. CT-values from RT-qPCRs were normalized to *B2M* and calculated as -fold ± S.E. (*p<0.05 TUSC3 *vs.* EV, t-test, n=3 per cell line).

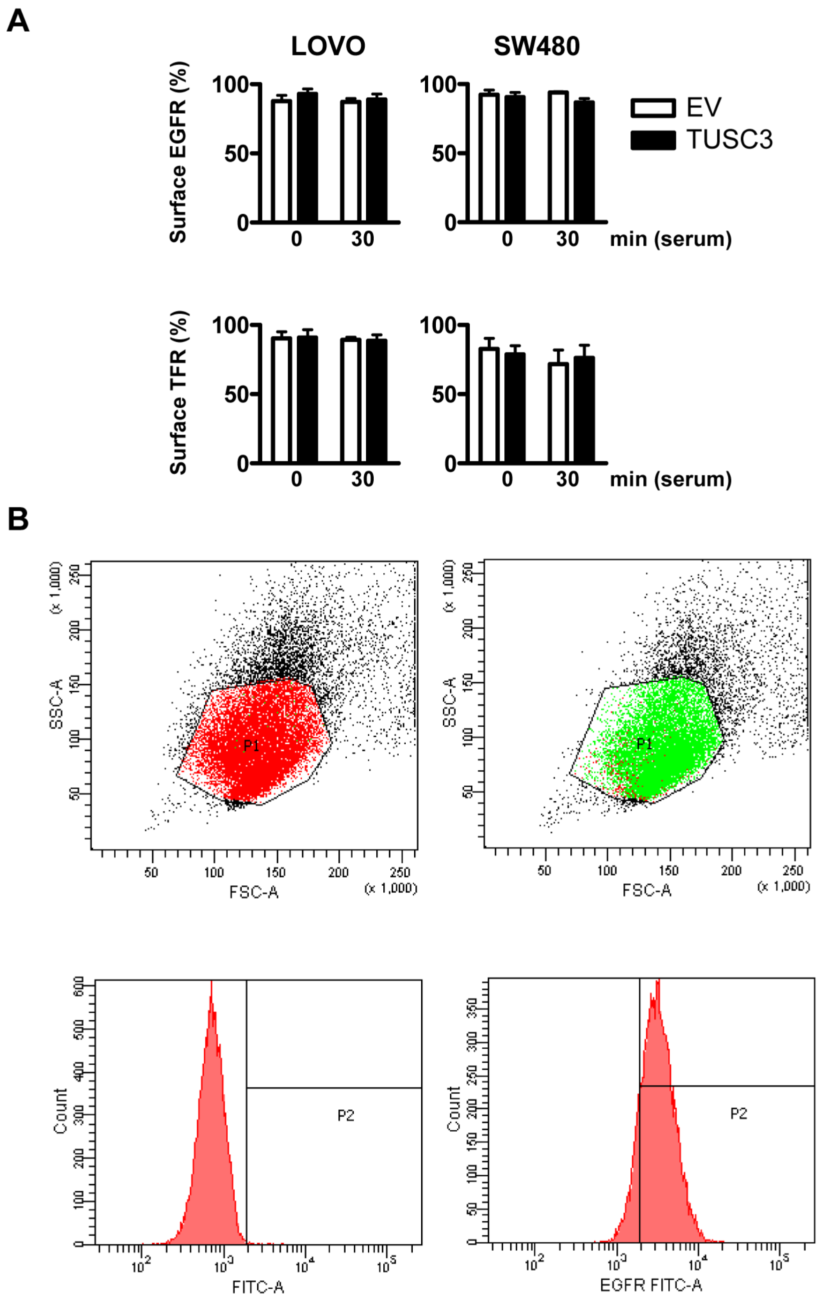

Supplementary Figure 6: TUSC3 has no effect on the amount of EGFR at the cell surface. SW480 and LOVO cells were transfected with TUSC3 or EV plasmids for 24 h, followed by serum removal ("starvation") for 16 h and a restimulation with 20 % FCS ("serum shock") for 0 to 30 min before cell harvest to evoke endocytosis of the EGFR. Cells were then subjected to fixation and staining with a FITC-labelled Ab against the N-terminal extracellular EGFR domain for flow cytometry (FC). Quantitative analyses (A) are shown together with (B) representative dot and intensity plots. Data are % receptor-positive cells ± S.E. (n.s. TUSC3 *vs.* EV, Two-way ANOVA, n=3-4 per cell line). Similar results were obtained for the transferrin receptor (TFR/CD71).

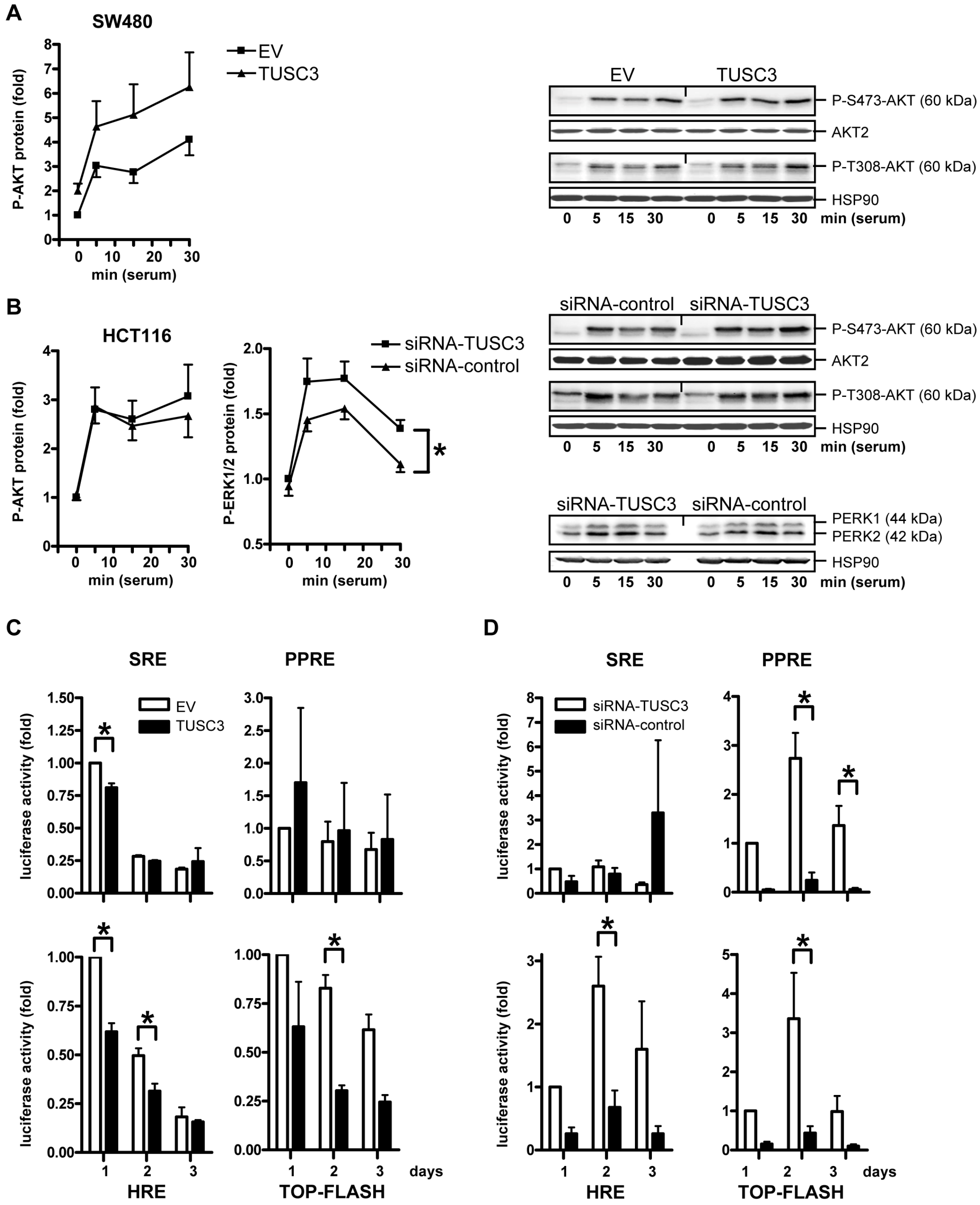

Supplementary Figure 7: TUSC3 inhibits downstream EGFR signaling. A, TUSC3 does not inhibit AKT phosphorylation. SW480 cells were transfected with TUSC3 or EV plasmids, then starved and stimulated as described in Fig.3A. Quantitation and representative Western blots are shown. Data are -fold ± S.E (n.s., TUSC3 *vs.* EV, Two-way ANOVA, n=3). B, TUSC3 knock-down increases ERK1/2 but not AKT phosphorylation. HCT116 cells were transfected with siRNAs and analyzed as in A (*p<0.05 siRNA-TUSC3 *vs.* siRNA-control, Two-way ANOVA, n=3). C-D, TUSC3 inhibits nuclear hypoxia and Wnt response. (C) HEK293T cells were transfected with TUSC3 or EV plasmids, (D) HCT116 cells with siRNAs together with luciferase reporter plasmids detecting hypoxia (HRE), mitogenic (SRE), differentiation (PPRE) or Wnt (TOP-FLASH) signaling responses. Luciferase activity was normalized to protein content and expressed as -fold ± S.E. (*p<0.05 TUSC3 *vs.* EV and siRNA-TUSC3 *vs.* siRNA-control, Two-way ANOVA, n=3 per plasmid or siRNA).

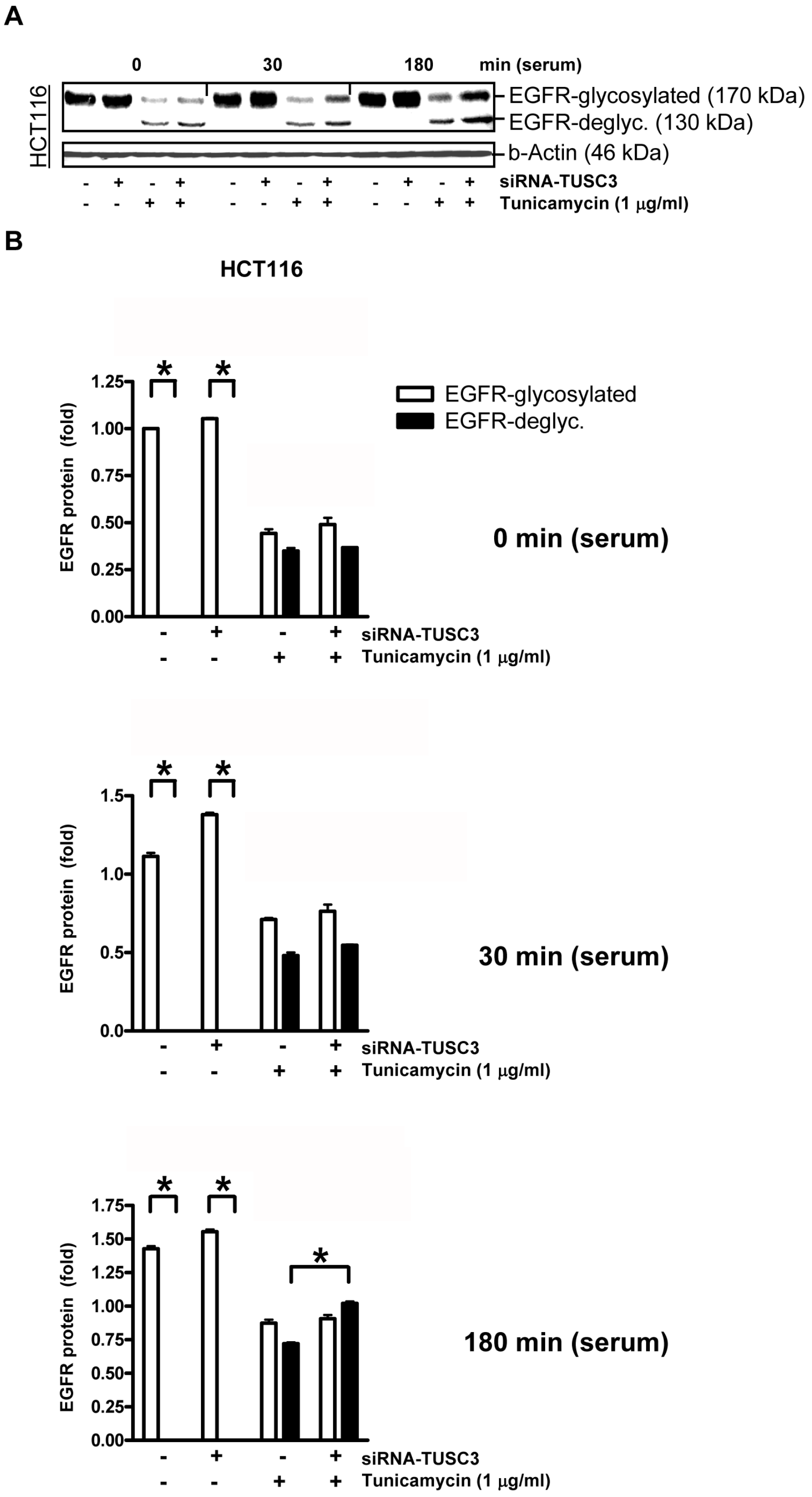

Supplementary Figure 8: Loss of TUSC3 augments tunicamycin-dependent EGFR deglycosylation. A, HCT116 cells were transfected with siRNAs for 6 h, followed by serum removal for 16 h in presence and absence of tunicamycin (1 µg/ml) and subsequent restimulation with serum (20 % FCS) for 0, 30 min to 3 h before extraction of TCL for Western blotting using the C-terminal EGFR Ab. Representative gels are shown. B, Quantitative analysis of Western blots in A. O.D. values from bands in gels are calculated as -fold ± S.E. (*p<0.05 EGFRp170 *vs*. EGFRp130, Two-way ANOVA, n=3).

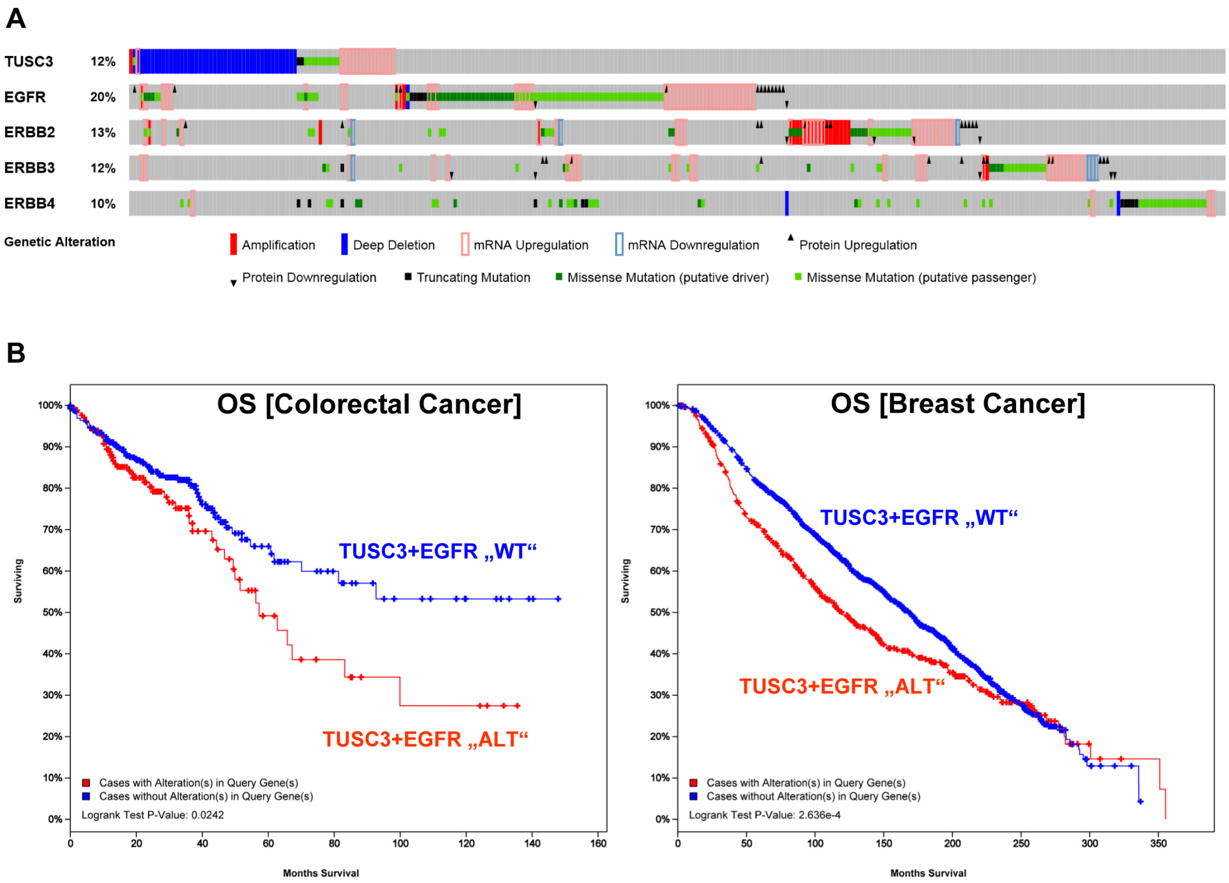

Supplementary Figure 9: Gene alterations in TUSC3 and EGFR predict poor prognosis in CRC patients. A, Overview of genetic changes in human *TUSC3* and *ERBB* genes. Oncoprint® files were retrieved from the cBioportal of Cancer Genomics data set: [Colorectal Adenocarcinoma, TCGA, Provisional (n=633 cases)]. Right: Patients without gene alterations were marked in grey or cut off. Left: Percent (%) altered cases compared with the total patient number. B, Kaplan-Meier survival analysis. The correlation of *TUSC3* and *EGFR* gene alterations to prognosis (Table S5) was calculated based on data sets from cBioportal of Cancer Genomics: [Colorectal Adenocarcinoma, TCGA, Provisional (n=633 cases); Breast Cancer, METABRIC, Nature 2012 & Nat Commun 2016 (n=2509 cases)]. Log rank tests are depicted in the graph.

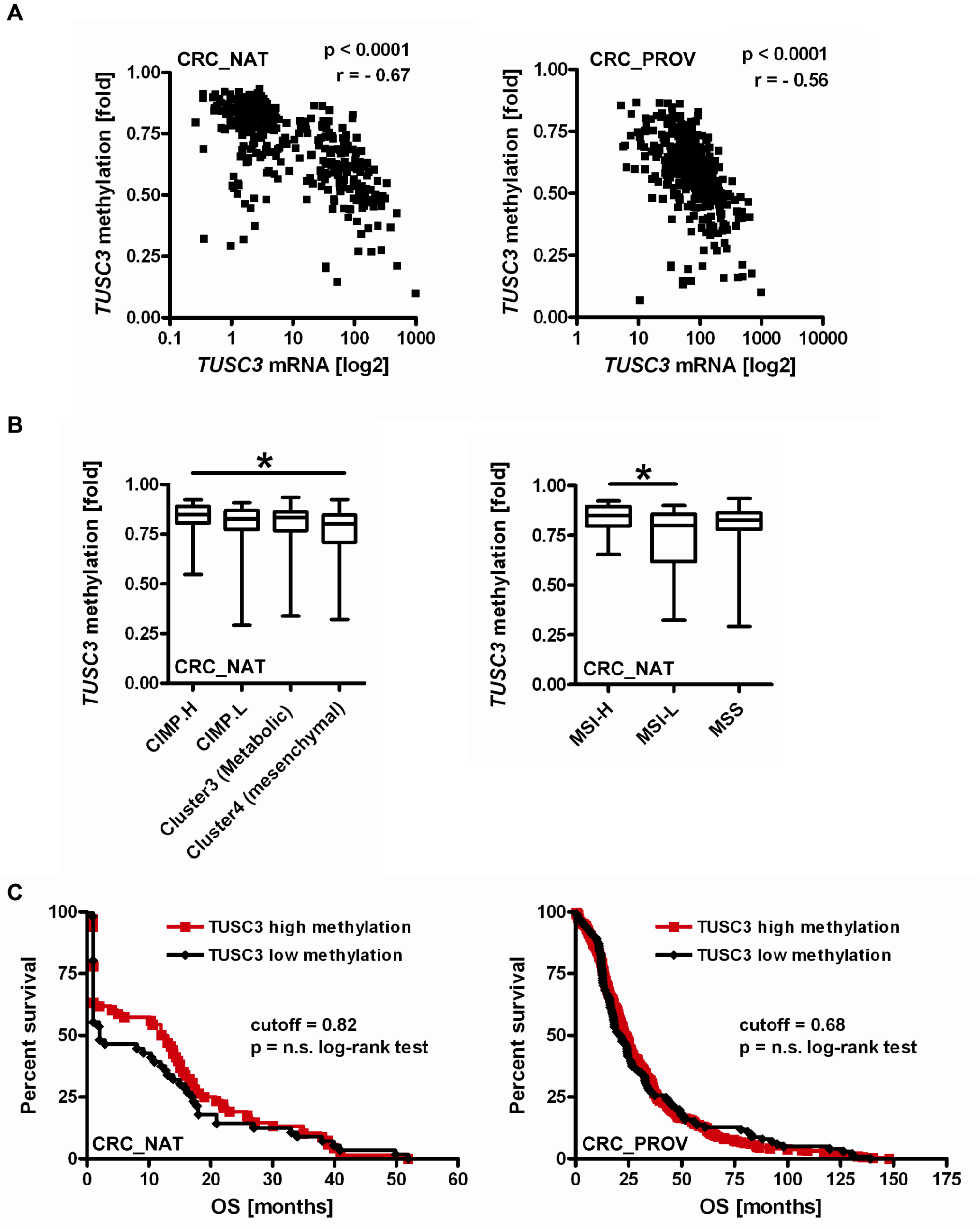

Supplementary Figure 10: Association of TUSC3 gene methylation with TUSC3 mRNA expression, molecular subtypes and prognosis in CRC patients. A, Correlation analysis was conducted based on two data sets from cBioportal of Cancer Genomics: [Colorectal Adenocarcinoma, TCGA, Provisional (n=633) and Nature 2012 (n=276)]. Spearman correlation coefficients and p-values are presented in the graphs. B, Box-Whisker plots showing increased *TUSC3* methylation in CRC subsets with CIMP-H and MSI-H status: [Colorectal Adenocarcinoma, Nature 2012 (n=276)] (*$p<0.05$, Kruskal Wallis test). C, Kaplan-Meier survival analysis for *TUSC3* methylation and overall survival (OS) using the data sets: [Colorectal Adenocarcinoma, TCGA, Provisional (n=633) and Nature 2012 (n=276)]. Cut-off values were determined by ROC analysis. Results from log rank test are shown within the graphs. Similar results were obtained for disease-free survival (DSS) (not shown).

For Supplementary Tables see in Supplementary Files.
