# Supplementary material for: Epigenetic silencing of tumor suppressor candidate 3 confers adverse prognosis in early colorectal cancer
Source: Oncotarget. 2017 Sep 15;8(49):84714–28. doi: 10.18632/oncotarget.20950 (PMC5689568; doi:10.18632/oncotarget.20950)
Supplement: Supplementary file 2 [file oncotarget-08-84714-s002.docx]

| **Table S1**  **Association of TUSC3 expression with prognosis in CRC patients.**  Total n=306 cases were dichotomized into TUSC3 low expression (score 1-2) and TUSC3 high expression (score 2-3): OS (overall survival). | | | | |
| --- | --- | --- | --- | --- |
| **Summary** | | | | |
| **TUSC3 positivity** | **Total cases** | **Events** | **Censored** | **Percent** |
| **0-1** | 71 | 26 | 45 | 63,4 |
| **2-3** | 182 | 48 | 134 | 73,6 |
| **Total** | 253 | 74 | 179 | 70,8 |
| **Mean OS in months** | | | | |
| **TUSC3 positivity** | **Estimate** | **SD** | **CI lower** | **CI upper** |
| **0-1** | 75,8 | 6,9 | 62,3 | 89,3 |
| **2-3** | 90,8 | 4,5 | 82,0 | 99,7 |
| **5-year survival as percentage** | | | | |
| **TUSC3 positivity** | **Estimate** | **SD** |  |  |
| **0-1** | 59,9 | 6,8 |  |  |
| **2-3** | 68,7 | 4,3 |  |  |
| **Pairwise comparison with log rank (Mantel-Cox)** | | | | |
| **TUSC3 positivity** | **Score 0-1** | | **Score 2-3** | |
|  | **Chi square** | **Sig.** | **Chi square** | **Sig.** |
| **0-1** |  |  | 2,174 | **0,140** |
| **2-3** | 2,174 | **0,140** |  |  |

| **Table S2**  **Association of TUSC3 expression with prognosis in CRC patient with N0 status.**  Total n=156 cases: OS (overall survival); N (nodal, invasion of local lymphnodes). | | | | |
| --- | --- | --- | --- | --- |
| **Summary (N0)** | | | | |
| **TUSC3 positivity** | **Total cases** | **Events** | **Censored** | **Percent** |
| **0-1** | 36 | 11 | 25 | 69,4 |
| **2-3** | 99 | 16 | 83 | 83,8 |
| **Total** | 135 | 27 | 108 | 80,0 |
| **Mean OS in months (N0)** | | | | |
| **TUSC3 positivity** | **Estimate** | **SD** | **CI lower** | **CI upper** |
| **0-1** | 82,6 | 9,7 | 63,6 | 101,6 |
| **2-3** | 108,4 | 4,6 | 99,5 | 117,4 |
| **5-year survival as percentage (N0)** | | | | |
| **TUSC3 positivity** | **Estimate** | **SD** |  |  |
| **0-1** | 62,7 | 10,1 |  |  |
| **2-3** | 85,4 | 4,1 |  |  |
| **Pairwise comparison (N0) with log rank (Mantel-Cox)** | | | | |
| **TUSC3 positivity** | **Score 0-1** | | **Score 2-3** | |
|  | **Chi square** | **Sig.** | **Chi square** | **Sig.** |
| **0-1** |  |  | 4,462 | *** 0,035** |
| **2-3** | 4,462 | *** 0,035** |  |  |

| **Table S3**  **Association of TUSC3 expression with prognosis in CRC patient with N+ status.**  Total n=140 cases: OS (overall survival); N (nodal, invasion of local lymphnodes). | | | | |
| --- | --- | --- | --- | --- |
| **Summary (N+)** | | | | |
| **TUSC3 positivity** | **Total cases** | **Events** | **Censored** | **Percent** |
| **0-1** | 35 | 15 | 20 | 57,1 |
| **2-3** | 83 | 32 | 51 | 61,4 |
| **Total** | 118 | 47 | 71 | 60,2 |
| **Mean OS in months (N+)** | | | | |
| **TUSC3 positivity** | **Estimate** | **SD** | **CI lower** | **CI upper** |
| **0-1** | 67,3 | 9,2 | 49,3 | 85,4 |
| **2-3** | 57,0 | 7,2 | 42,8 | 71,1 |
| **5-year survival as percentage (N+)** | | | | |
| **TUSC3 positivity** | **Estimate** | **SD** |  |  |
| **0-1** | 57,7 | 8,8 |  |  |
| **2-3** | 39,7 | 8,6 |  |  |
| **Pairwise comparison (N+) with log rank (Mantel-Cox)** | | | | |
| **TUSC3 positivity** | **Score 0-1** | | **Score 2-3** | |
|  | **Chi square** | **Sig.** | **Chi square** | **Sig.** |
| **0-1** |  |  | 0,023 | 0,880 |
| **2-3** | 0,023 | 0,880 |  |  |

| **Table S4**  **Association of TUSC3 expression with CRC clinical factors.**  Total n = 306 cases. | | | |
| --- | --- | --- | --- |
| **TUSC3 positivity** | **0-1 (low)** | **2-3 (high)** | **Total n [%]** |
| **Gender** |  |  |  |
| **Male** | 49 [30] | 117 [70] | 166 [100] |
| **Female** | 43 [31] | 97 [69] | 140 [100] |
| **Total** | 92 [30] | 214 [70] | 306 [100] |
| **[Chi Squ.] Sig** | [0,052] 0,900 | | |
| **Age** |  |  |  |
| <**67 y** | 43 [28] | 110 [72] | 153 [100] |
| >**67 y** | 49 [32] | 104 [68] | 153 [100] |
| **Total** | 92 [30] | 214 [70] | 306 [100] |
| **[Chi Squ.] Sig** | [0,560] 0,533 | | |
| **Localization** | | | |
| **Coecum** | 9 [33] | 18 [67] | 27 [100] |
| **Ascencens** | 23 [40] | 34 [60] | 57 [100] |
| **Flexura dextra** | 2 [22] | 7 [78] | 9 [100] |
| **Transversum** | 11 [41] | 16 [59] | 27 [100] |
| **Flexura sinistra** | 4 [29] | 10 [71] | 14 [100] |
| **Descendens** | 5 [33] | 10 [67] | 15 [100] |
| **Sigma** | 21 [28] | 53 [72] | 74 [100] |
| **Rektosigmoid** | 1 [10] | 9 [90] | 10 [100] |
| **Rectum** | 13 [21] | 48 [79] | 61 [100] |
| **Not known** | 1 [25] | 3 [75] | 4 [100] |
| **Multifocal** | 2 [25] | 6 [75] | 8 [100] |
| **Total** | 92 [30] | 214 [70] | 306 [100] |
| **[Chi Squ.] Sig** | [9,207] 0,518 | | |
| **T** | | | |
| **T1** | 1 [9] | 10 [91] | 11 [100] |
| **T2** | 14 [27] | 37 [73] | 51 [100] |
| **T3** | 66 [34] | 130 [66] | 196 [100] |
| **T4** | 11 [23] | 37 [77] | 48 [100] |
| **Total** | 92 [30] | 214 [70] | 306 [100] |
| **[Chi Squ.] Sig** | [4,847] 0,649 | | |
| **N** | | | |
| **N0** | 46 [29] | 114 [71] | 160 [100] |
| **N1** | 23 [31] | 51 [69] | 74 [100] |
| **N2** | 23 [32] | 49 [68] | 72 [100] |
| **Total** | 92 [30] | 214 [70] | 306 [100] |
| **[Chi Squ.] Sig** | [0,289] 0,855 | | |
| **M** | | | |
| **M0** | 78 [29] | 191 [71] | 269 [100] |
| **M1** | 14 [38] | 23 [62] | 37 [100] |
| **Total** | 92 [30] | 214 [70] | 306 [100] |
| **[Chi Squ.] Sig** | [1,209] 0,339 | | |
| **L** | | | |
| **L0** | 42 [29] | 105 [71] | 147 [100] |
| **L1** | 50 [31] | 109 [69] | 159 [100] |
| **Total** | 92 [30] | 214 [70] | 306 [100] |
| **[Chi Squ.] Sig** | [0,300] 0,619 | | |
| **V** | | | |
| **V0** | 73 [29] | 181 [71] | 254 [100] |
| **V1** | 19 [37] | 32 [63] | 51 [100] |
| **Total** | 92 [30] | 213 [70] | 305 [100] |
| **[Chi Squ.] Sig** | [0,1462] 0,244 | | |
| **R** | | | |
| **R0** | 89 [30] | 206 [70] | 295 [100] |
| **R1** | 3 [27] | 8 [73] | 11 [100] |
| **Total** | 92 [30] | 214 [70] | 306 [100] |
| **[Chi Squ.] Sig** | [0,042] 1,000 | | |
| **G** | | | |
| **G1** | 0 [0] | 8 [100] | 8 [100] |
| **G2** | 61 [30] | 139 [70] | 200 [100] |
| **G3** | 31 [32] | 67 [68] | 98 [100] |
| **Total** | 92 [30] | 214 [70] | 306 [100] |
| **[Chi Squ.] Sig** | [3,572] 0,184 | | |

| **Table S5 Correlation of *TUSC3* and *ERBB* gene alterations with prognosis in human cancers.** Kaplan-Meier analysis based on cBioPortal genomic data bases: Colorectal Adenocarcinoma, TCGA, Provisional (n=633) abbrev. CRC_PROV; Breast Cancer, METABRIC, Nature 2012 & Nat Commun 2016 (n=2509) abbrev. BC_METABRIC; * cases with alterations (“ALT“); # cases without alterations (“WT”); § not assessable (“NA”);  OS overall survival; DFS disease-free survival [not shown, n.s. p>0.05 for all data bases]. | | | | | |
| --- | --- | --- | --- | --- | --- |
| **gene(s)** | **status** | **total cases** | **cases deceased** | **median month**  **OS** | **log-rank test**  **p-value** |
| **CRC_PROV** | | | | | |
| **TUSC3** | | | | | |
|  | ALT * | 72 | 16 | 65.8 | 0.562 |
|  | WT # | 547 | 113 | 92.67 |  |
| **EGFR** | | | | | |
|  | ALT | 125 | 36 | 57.19 | *** 0.0403** |
|  | WT | 495 | 93 | NA & |  |
| **ERBB2** | | | | | |
|  | ALT | 83 | 20 | 99.93 | 0.468 |
|  | WT | 537 | 109 | 83.18 |  |
| **ERBB3** | | | | | |
|  | ALT | 76 | 12 | 65.8 | 0.426 |
|  | WT | 543 | 117 | 83.18 |  |
| **ERBB4** | | | | | |
|  | ALT | 65 | 16 | 99.93 | 0.338 |
|  | WT | 554 | 113 | 83.18 |  |
| **TUSC3 EGFR** | | | | | |
|  | ALT | 178 | 48 | 57.19 | *** 0.0242** |
|  | WT | 442 | 81 | NA |  |
| **TUSC3 ERBB2** | | | | | |
|  | ALT | 143 | 34 | 67.25 | 0.238 |
|  | WT | 477 | 95 | 92.67 |  |
| **TUSC3 ERBB3** | | | | | |
|  | ALT | 141 | 27 | 65.8 | 0.887 |
|  | WT | 478 | 102 | 92.67 |  |
| **TUSC3 ERBB4** | | | | | |
|  | ALT | 127 | 30 | 65.8 | 0.307 |
|  | WT | 492 | 99 | 92.67 |  |
| **BC_METABRIC** | | | | | |
| **TUSC3** | | | | | |
|  | ALT | 105 | 73 | 139.63 | 0.152 |
|  | WT | 1875 | 1070 | 157.83 |  |
| **EGFR** | | | | | |
|  | ALT | 211 | 126 | 120.13 | *** 0.0361** |
|  | WT | 1769 | 1017 | 159.7 |  |
| **ERBB2** | | | | | |
|  | ALT | 445 | 259 | 118.6 | *** 0.00112** |
|  | WT | 1535 | 884 | 165.4 |  |
| **ERBB3** | | | | | |
|  | ALT | 149 | 86 | 145.43 | 0.289 |
|  | WT | 1831 | 1057 | 156.8 |  |
| **ERBB4** | | | | | |
|  | ALT | 119 | 81 | 126.66 | 0.299 |
|  | WT | 1861 | 1062 | 158.63 |  |
| **TUSC3 EGFR** | | | | | |
|  | ALT | 298 | 187 | 135.33 | *** 0.0211** |
|  | WT | 1682 | 956 | 161.13 |  |
| **TUSC3 ERBB2** | | | | | |
|  | ALT | 526 | 316 | 119.46 | *** 2.636e-4** |
|  | WT | 1454 | 827 | 168.33 |  |
| **TUSC3 ERBB3** | | | | | |
|  | ALT | 248 | 157 | 142.43 | 0.0516 |
|  | WT | 1732 | 986 | 159.23 |  |
| **TUSC3 ERBB4** | | | | | |
|  | ALT | 204 | 136 | 128.7 | 0.0496 |
|  | WT | 1776 | 1007 | 159.7 |  |

| **Table S6 Oligonucleotides**  BSP (Bisulfite Sequencing), cDNA (for cloning of CDS), CDS (Protein coding sequence), HRM (High Resolution Melting analysis), ML (MethylLight PCR), NGS (Next Generation Sequencing), RT-PCR (Reverse Transcription PCR) | | | | |
| --- | --- | --- | --- | --- |
| **Gene** | **Sequence (5’>3’)** | **Product length** | **Accession Nr.** | **Method** |
| **B2M** |  |  | **NM_004048** | **RT-PCR** |
| **F** | TGCTGTCTCCATGTTTGATGTATCT | **85 bp** |  |  |
| **R** | TCTCTGCTCCCCACTCTAAGT |  |  |  |
| **TUSC3** |  |  |  | **RT-PCR** |
| **F** | GCAGCTGATGGAATGGAGTT | **400 bp** | **NM_006765.3**  Exon 2-4 |  |
| **R** | ATCCGTTCTGTCAGCAATCC |  | **NM_178234.2**  Isoforms 1 and 2 |  |
| **TUSC3** |  |  | **NG_012141.2** | **ML/HRM** |
| **F** | CCGAACAAACGTAATACGCG | **105 bp** | CpG Island  Exon 1  **5136-5240 bp** |  |
| **R** | ACGGCGTGAAGGAGCG |  |  |  |
| **Probe** | TACGCGCGGTAGTCGTGCGC |  | 3'BHQ1-5'FAM |  |
| **TUSC3** |  |  | **NG_012141.2** | **BSP/HRM** |
| **F** | *TAGATTGA*GGTTTTAGGGTTAAAGGATTAT | **451 bp** | CpG Island  Promoter/Exon 1  **4866-5316 bp** |  |
| **R** | TACAAAACAACAACAACAAAAAAAA |  |  |  |
| **TUSC3** |  |  | **NG_012141.2** | **BSP** |
| **F** | AGGATGGTTT*TAGATTGA*GGTTTTAGG | **511 bp** | CpG Island  Promoter/Exon 1  **4856-5366 bp** |  |
| **R** | CAAAAAAATCCATTCTACCTCCTTTTT |  |  |  |
| **TUSC3** |  |  | **NG_012141.2** | **NGS** |
| **F** | GGTTTTAGGGTTAAAGGATTA | **335 bp** | CpG Island  Promoter/Exon 1  **4874-5208 bp** |  |
| **R** | C**R**ACAAAACAATATCTCCTC |  |  |  |
| **TUSC3** |  |  | **NM_006765.3** | **cDNA** |
| **F** | AGGAGACACTGCCCTGCC | **1100 bp** | **324-1423 bp**  Exon 1-10 | CDS isoform 1 |
| **R** | TTTTTAAGTGCCATGGTCCAA |  |  |  |
| **TUSC3** |  |  | **NM_178234.2** | **cDNA** |
| **F** | AGACACTGCCCTGCCGCGAT | **1075 bp** | **327-1401 bp**  Exon 1-11 | CDS isoform 2 |
| **R** | ATCCCACTTGGCTTCATTTA |  |  |  |
